# Nanosized Drug Delivery Systems to Fight Tuberculosis

**DOI:** 10.3390/pharmaceutics15020393

**Published:** 2023-01-24

**Authors:** Tom Bourguignon, Jesus Alfredo Godinez-Leon, Ruxandra Gref

**Affiliations:** Institut des Sciences Moléculaires d’Orsay, Université Paris-Saclay, CNRS, 91405 Orsay, France

**Keywords:** nanoparticles, drug nanocarriers, drug delivery systems, targeted delivery, host-directed therapy, physicochemical characterization, biodegradable polymers, tuberculosis, antitubercular treatment

## Abstract

Tuberculosis (TB) is currently the second deadliest infectious disease. Existing antitubercular therapies are long, complex, and have severe side effects that result in low patient compliance. In this context, nanosized drug delivery systems (DDSs) have the potential to optimize the treatment’s efficiency while reducing its toxicity. Hundreds of publications illustrate the growing interest in this field. In this review, the main challenges related to the use of drug nanocarriers to fight TB are overviewed. Relevant publications regarding DDSs for the treatment of TB are classified according to the encapsulated drugs, from first-line to second-line drugs. The physicochemical and biological properties of the investigated formulations are listed. DDSs could simultaneously (i) optimize the therapy’s antibacterial effects; (ii) reduce the doses; (iii) reduce the posology; (iv) diminish the toxicity; and as a global result, (v) mitigate the emergence of resistant strains. Moreover, we highlight that host-directed therapy using nanoparticles (NPs) is a recent promising trend. Although the research on nanosized DDSs for TB treatment is expanding, clinical applications have yet to be developed. Most studies are only dedicated to the development of new formulations, without the in vivo proof of concept. In the near future, it is expected that NPs prepared by “green” scalable methods, with intrinsic antibacterial properties and capable of co-encapsulating synergistic drugs, may find applications to fight TB.

## 1. Introduction

Almost a third of the world’s population is infected with *Mycobacterium tuberculosis* (*Mtb*), and is therefore at risk of developing an active form of tuberculosis (TB) [1,2]. With an incidence of ten million cases and between one and two million deaths each year, *Mtb* was the second deadliest infectious agent in 2021 after SARS-CoV-2. Moreover, the recent coronavirus pandemic affected public health services in such a way that for the first time since 2005, the mortality rate associated with *Mtb* increased [3]. However, TB is not a recent threat to humanity.

**Figure 1:**
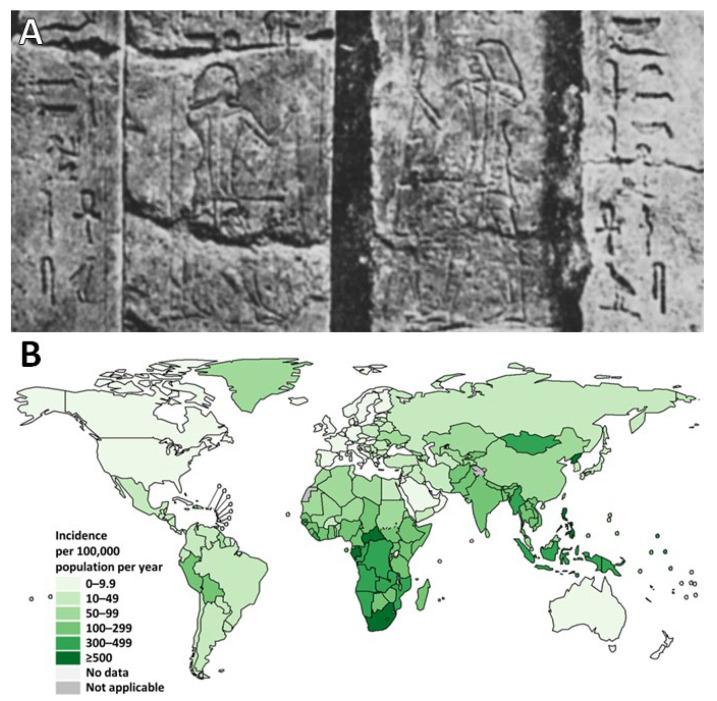
(**A**) Engravings on the doors of an Egyptian tomb depicting individuals suffering from tuberculosis (TB). The arm is exaggeratedly contorted to emphasize the deformity. Adapted from reference [6], copyright (2022) by Copyright Clearance Center, with permission from Elsevier. (**B**) Estimated TB incidence rates over the world in 2021. Adapted from reference [9], used under Creative Commons BY-NC-SA 3.0 IGO license.

The genus *Mycobacterium* is over one hundred and fifty million years old. The first ancestor of *Mtb* appeared in East Africa three million years ago, while the common ancestor of current strains would be between fifteen and twenty thousand years old [4,5]. The first documented clinical case of TB is not found in literature, but in art: indeed, three thousand years B.C., in ancient Egypt, were engraved drawings on the doors of tombs representing deformed men and women, suffering from protuberances in the back [6,7] (Figure 1A). The individuals depicted on these monuments are likely to have suffered from Pott’s disease, an infection of the vertebral bodies due to the dissemination of *Mtb*. In 1998, Crubézy et al. identified *Mycobacterium* DNA on a 5400-year-old Egyptian mummy, thus confirming that TB has been the scourge of the human race since ancient times, claiming countless number of lives [8].

For millennia, no effective treatment against *Mtb* was found, until the 19th century: in 1854, Hermann Brehmer opened the first sanatorium, which produced unprecedented results, surpassing all other treatments of that time [10]. In 1865, Jean-Antoine Villemin demonstrated the infectious nature of the disease [11], and in 1882, Robert Koch discovered and isolated the pathogen, which then took the name of Koch’s bacillus [12]. Finally, in 1919, Albert Calmette and Camille Guérin developed BCG (bacillus Calmette–Guérin), the only vaccine to date used to combat the epidemic, first administered to humans in 1921 [13]. During the decades that followed, antitubercular drugs were discovered (beginning with streptomycin in 1945), including those which still constitute the first-line treatment today: isoniazid (INH) (1951), ethambutol (EMB) (1961), rifampicin (RFP) (1966), and pyrazinamide (PZA) (discovered in the late 1940s, but used much later in combination with INH and RFP) [14].

However, although the general opinion regards TB as a disease of the past in developed countries, *Mtb* still remains a major health problem in emerging countries (Figure 1B). One of the goals of the World Health Organization (WHO) was to “end the epidemic” by 2035, reducing the mortality rate by 95%. However, after two decades of progress regarding the testing and diagnosis of newly infected patients, the 2020 coronavirus pandemic hindered a dynamic which will take additional years of work to relaunch. Kirby reported that in 2020, the number of new diagnoses fell by almost 20% compared to 2019 (from 7.1 million to 5.8 million), a figure which McQuaid et al. estimated to be as high as 30% [15,16]. As a consequence, in 2021, and for the first time in nearly twenty years, the number of deaths linked to TB increased (by 1.5 million, compared to 1.4 million in 2020) [15].

Humanity is and will be facing new epidemics in the future, but biomedical research is also gaining access to new therapeutics. In that regard, 2020 was the advent of a new era, with the introduction on the market of the first mRNA vaccine [17]. Research on mRNA as an active substance began in the 1980s. Very quickly, the question arose of not only transporting the active ingredient to the targeted cells, but also of protecting it from degradation in the physiological environment [18]. Hence, lipid nanoparticles (NPs) played a crucial role during the pandemic, constituting the important innovation which enabled the design of mRNA vaccines against SARS-CoV-2.

NPs meet multiple objectives (mainly protection and vectorization of active ingredients, controlled and prolonged release, reducing doses and toxic side effects) and are therefore attracting more and more interest over time. However, their impact on the market is still moderate. Currently, around fifty products comprising nanovectors are approved by the Food and Drug Administration (FDA), and around fifty others are going through clinical trials [19]. Regarding TB, the prospects offered by NPs are more than promising, but the question here is to understand how they could integrate the current therapeutic arsenal. In the first part of this review, TB and its treatment will be presented. Then, the diversity and characteristics of the main types of drug nanocarriers will be covered. Subsequently, a review will be made of nanosized DDSs that carry drugs for TB treatment. Finally, other trends, such as host-directed therapies and new methods to treat TB, will be highlighted.

## 2. Tuberculosis

### 2.1. Physiopathology

Rethinking and optimizing the treatment of TB requires a general understanding of its physiopathology. Before focusing on the behavior of the pathogen at the cellular level, it is necessary to comprehend how *Mtb* operates at the level of the entire organism. TB is transmitted by airborne droplets emitted by an individual suffering from an active form of the disease. During infection, the subject inhales bacilli, most of which are mechanically retained by mucus in the upper respiratory tract (usually with diameters higher than 5 µm) [20]. However, a small fraction (around 10%) reaches the lungs, bronchioles and alveoli, where alveolar macrophages then capture the bacteria [21]. At this stage, around 70% of subjects manage to eliminate the pathogen through the innate immune response. Otherwise, alveolar macrophages cross the lung epithelium to reach the interstitium [22].

**Figure 2:**
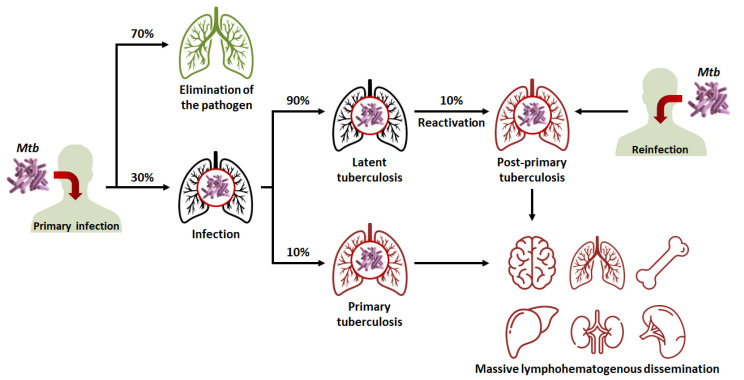
Evolution of the different clinical stages of tuberculosis (TB), from primary infection to miliary TB. *Mtb*: *Mycobacterium tuberculosis*.

In order to contain the infection, a characteristic cellular structure is then formed, typical of TB: the granuloma. The granuloma is a complex set of immune and inflammatory cells (such as macrophages, neutrophils, fibroblasts, T lymphocytes, B lymphocytes) that surrounds the infectious site and produces various cytokines and chemokines in order to maintain macrophage activation [23,24]. The purpose of the granuloma is not only to contain the proliferation of *Mtb*, but also to prevent its dissemination to other organs. What happens next is determined by the subject’s immunocompetence (Figure 2):

- In 90% of cases, the granuloma, acting as a physical and immunological barrier will succeed in stemming the infection. The pathogen will be contained in necrotic areas within granulomas located in the lungs [25]. Fibrous lesions will develop, and TB will evolve towards a latent form. This explains the fact that almost a third of the world’s population carries the pathogen, but that not more than ten million cases of active TB are diagnosed every year. Thus, most subjects that are latent cases (90%) will carry *Mtb* for decades, but will not show symptoms or infect other individuals [22].
- In 10% of cases, most often in very young, elderly, or immunocompromised subjects, the granuloma will fail to contain *Mtb*. This phenomenon can also occur after the reactivation of dormant bacilli (which happens in 10% of latent cases) or with a novel inhalation of bacilli. The adaptive immune response will attempt to eliminate bacteria multiplying and escaping macrophages, but in doing so, will cause the destruction of lung tissue [1,26]. This will lead to the formation of caseous lesions and cavities, within which the growth of *Mtb* will no longer be controlled. TB will then evolve towards an active form. In certain cases, the pathogen will spread to other organs (brain, bones, liver, spleen, kidneys), and, in the most serious cases, the disease will evolve towards a miliary form, involving the massive lymphohematogenous dissemination of bacteria [27].

There is thus a balance between the latent form of TB, where the host’s immune system protects the subject while *Mtb* remains dormant within granulomas; and the active form, where *Mtb* proliferates to the detriment of its host. Thus, Koch’s bacillus is the “perfect” pathogen, in the sense that it preserves its host long enough for its own persistence while spreading through the population. In order to fully understand the success of *Mtb*’s “strategy”, it is necessary to study its behavior at the intracellular level. As previously said, after the inhalation of bacilli, the latter are phagocytosed by alveolar macrophages. Under physiological conditions, phagocytosis involves the acidification of the intracellular vesicle and its fusion with the lysosome (which allows its content to be degraded).

However, *Mtb* escapes the immune system by secreting various proteins which hinder the different stages of phagosomal maturation. Of note, it prevents the recruitment of GTPases and v-ATPases to the phagosomal membrane [23,24]. It also possesses a secretion system (ESX-1) that damages and permeabilizes the phagosomal membrane, which, in some cases, allows it to escape into the cytosol [28,29]. Thus, *Mtb* hijacks the defenses of its host to establish a niche inside of which it can either remain dormant or replicate, until causing the apoptosis or necrosis of the cell to disseminate germs and infect other cells [30].

### 2.2. Treatment

**Figure 3:**
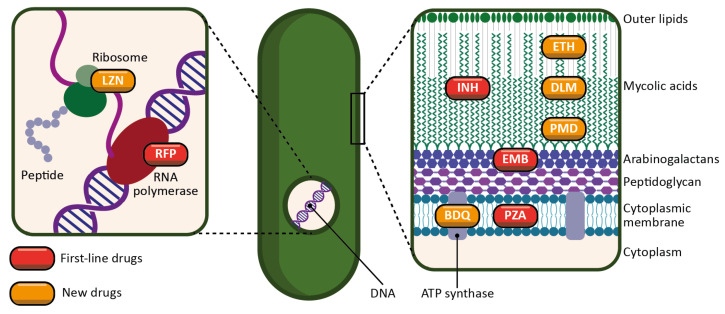
Schematic representation of the targets and mechanisms of action of the main antitubercular drugs. First-line drugs are represented in red (INH: isoniazid; RFP: rifampicin; PZA: pyrazinamide; EMB: ethambutol). New drugs, either newly discovered or repurposed, are represented in orange (ETH: ethionamide; LZN: linezolid; BDQ: bedaquiline; DLM: delamanid; PMD: pretomanid).

Thanks to the effectiveness of the innate immune response, there have always been survivors of TB. However, the introduction of antibiotics to the market was a revolution which considerably reduced mortality rates over time [31]. As mentioned in the introduction, the discovery of the four first-line antitubercular antibiotics dates back more than half a century. Here, we briefly present their mechanisms of action (Figure 3):

- INH is a prodrug activated by a bacterial enzyme (KatG) [32]. The activation of the molecule produces an inhibitor of another bacterial enzyme, InhA, which results in the inhibition of mycolic acid synthesis, and therefore of the bacterial wall.
- RFP is an inhibitor of the bacterial RNA polymerase, and thus acts by preventing protein synthesis [33]. It inhibits the elongation of bacterial RNA once it reaches two to three nucleotides in length.
- PZA is a prodrug metabolized by a bacterial enzyme (pyrazinamidase) to become pyrazinoic acid [34]. The exact mechanism of action of pyrazinoic acid is still only partially elucidated, but the molecule is thought to act simultaneously on membrane energy production, the ribosomal protein RpsA, and other yet unidentified bacterial targets.
- EMB targets arabinosyl transferase (a bacterial enzyme), thereby inhibiting arabinogalactan and bacterial wall synthesis [35]. EMB is also thought to exert a synergistic effect on INH activity.

The first-line treatment of TB is so effective that, in the case of a non-resistant strain and a therapy followed to completion, the risk of relapse is only 5%–8% [36,37]. Associated with INH, RFP reduces the duration of the therapy from eighteen to nine months. Taken for the first two months, PZA further reduces its duration by three months, leaving it at six months. EMB, finally, is used as an additional precaution in the event of unidentified resistance to one of the three main antibiotics. Thus, the treatment begins with a two-month induction phase involving the daily oral intake of the four antitubercular drugs. At the end of this phase, for most patients, no cultivable bacteria can be found in the sputum. The induction phase is followed by a four-month consolidation phase involving only INH and RFP to avoid possible relapse. Although there is considerable evidence of this treatment’s effectiveness, it can be prolonged and become more complex in the case of drug-resistant strains [38].

**Figure 4:**
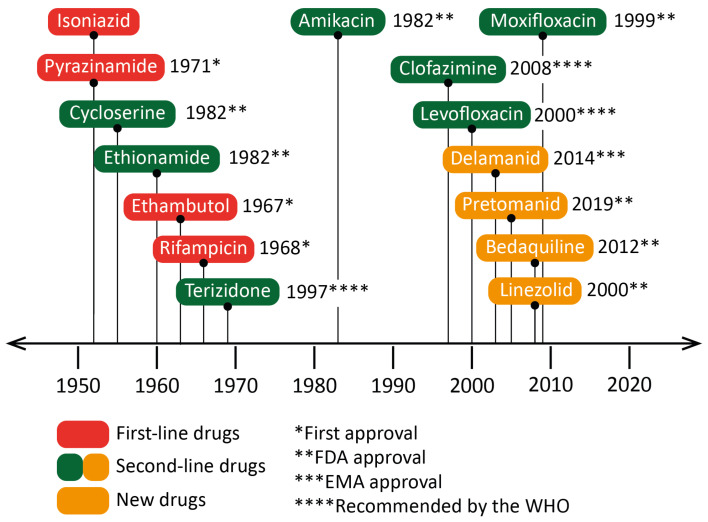
Dates of the first clinical trials for drugs recommended by the World Health Organization (WHO) for the treatment of tuberculosis (TB). The year of approval (or recommendation) for the use against *Mycobacterium tuberculosis* or against bacteria of the *Mycobacterium* genus is indicated next to the drug’s name. Isoniazid was accepted for prescription shortly after its first clinical trials. Some drugs, such as linezolid or moxifloxacin, were approved as broad-spectrum antibiotics, although specific clinical trials against TB occurred later. EMA: European Medicines Agency.

Drug resistance is a complex phenomenon involving an interplay of clinical, biological and microbiological processes [39]. In addition to the intrinsic resistance of bacteria, the lack of treatment adherence of patients also leads to the emergence of genetic resistance. Moreover, the complexity of granulomas is a barrier to the effective distribution of drugs, and therefore restrains their adequate supply. All this limits the use of first-line drugs and requires new antibiotics for therapy. There is a wide variety of drugs, usually used in combination, to treat cases of antibiotic-resistant TB. However, in the last twenty years, only four new drugs have been approved for this purpose: linezolid (LZN), bedaquiline (BDQ), delamanid (DLM) [39,40], and more recently, pretomanid (PMD) [41] (Figure 4). The mechanisms of action of some second-line drugs are described below (Figure 3):

- Ethionamide (ETH) is a thioisonicotinamide with a structure similar to that of INH [42]. ETH is a prodrug that, like INH, must be activated in order to inhibit mycobacterial fatty acid synthesis (by inhibiting enoyl-ACP reductase), which is essential for the production and repair of the bacterial cell wall.
- LZN is a synthetic antimicrobial drug of the oxazolidinone class [43]. By binding to the rRNA on the 50S and 30S ribosomal subunits, it blocks the synthesis of bacterial proteins.
- BDQ is the only FDA-approved antitubercular drug that targets the production of ATP [44]. BDQ inhibits the proton pumping mechanism by binding to the c subunit of the ATP synthase complex. It has also been observed that BDQ is able to act on the ε subunit of the enzyme.
- DLM is a prodrug that, like INH, prevents the synthesis of mycolic acid in the bacterial cell wall [45]. DLM inhibits the synthesis of methoxy- and keto-mycolic acid by acting on the mycobacterial F420 system.
- PMD is also a prodrug that acts under different mechanisms [46]. Under aerobic conditions, PMD inhibits protein and lipid synthesis by decreasing the availability of keto-mycolic acids through the inadequate oxidative transformation of the hydroxymycolate precursor. Under anaerobic conditions, PMD generates desnitro metabolites and provokes the release of nitric oxide, which inhibits cytochrome c oxidase and leads to a significant reduction in the amount of ATP present in bacteria.

In December 2022, the WHO released an update for the treatment of drug-resistant TB [47]. However, before addressing the new recommendations, it is necessary to define certain types of TB that are mentioned in the guidelines:

- Multidrug-resistant TB (MDR-TB) is defined as TB with an RFP-resistant (RR-TB) and INH-resistant strain.
- MDR/RR-TB stands for either MDR-TB or RR-TB.
- Pre-extensively drug-resistant TB (pre-XDR-TB) is defined as MDR/RR-TB with resistance to at least one fluoroquinolone (either levofloxacin (LVX) or moxifloxacin (MOX)).
- Extensively drug-resistant TB (XDR-TB) is defined as MDR/RR-TB with resistance to at least one fluoroquinolone (either LVX or MOX) and to at least one of the following two drugs: LZN and BDQ.
- Thus, the updated recommendations are as follows:
- For an INH-resistant only strain, the treatment is continued with RFP, PZA and EMB for a period of six months. INH is replaced by LVX.
- For MDR/RR-TB and pre-XDR-TB:
  - Firstly, the WHO suggests adopting a six-month regimen (BPaLM) comprising LZN, BDQ, PMD and MOX (in the absence of a MOX-resistant strain). It is urged to use this new regimen instead of the nine-month or longer regimens for MDR/RR-TB, since BPaLM provides superior results in a shorter period.
  - Secondly, for MDR/RR-TB without a resistance to fluoroquinolones, the WHO recommends using a nine-month regimen rather than longer (eighteen-month) regimen. This regimen consists of BDQ (for six months) in combination with a fluoroquinolone (LVX or MOX), INH, PZA, EMB, ETH and clofazimine (CFZ) (for four months, with the possibility of extending this period to six months if the patient remains sputum smear-positive after four months), and then, a fluoroquinolone (LVX or MOX), PZA, EMB and CFZ (for five months). Two months of LZN might be used as an alternative to ETH.
- For XDR-TB, the complementary molecules mentioned above constitute the core of the treatment.

Although the treatment of TB is theoretically effective, it is long, constraining and costly, even more in the case of antibiotic resistance (which concerns 20%–25% of clinical cases) [48]. However, it is precisely the complexity of the therapy that impedes patient compliance, and ultimately favors the emergence of resistant strains. In general, antitubercular drugs have low solubility, low metabolic stability, and low tissue penetration [49]. The challenge is to achieve therapeutic concentrations at the site of infection. The case of TB is troublesome, since multiple biological barriers stand in the way of active substances. Not only must the latter be directed towards the organ of interest (most often the lungs), but they must also cross the granulomas, the host cell membranes, and finally, the cell membranes of the pathogen hidden within infected cells [50].

Since the treatment is administered orally and diffuses systemically in the organism, high doses are required to reach sufficient concentrations at the site of infection. However, this therapy generates a strong accumulation of active molecules in non-targeted organs, and thus increases the side effects associated with these molecules. In 2019, Prasad et al. listed the side effects of antitubercular drugs, on the basis of more than a hundred articles and studies [51]. The first finding is that toxicity is more likely to occur during the induction phase rather than during the consolidation phase (which is expected, since the former involves more active molecules than the latter). The second finding is that first-line antibiotics present less toxicity (prevalence in 8%–85% of clinical cases) than second-line antibiotics (prevalence in 69%–96% of clinical cases). The third observation is that most of the side effects (gastrointestinal disorders, hepatotoxicity, peripheral neuropathy, optic neuritis, ototoxicity, nephrotoxicity, and skin reactions) are minor if they are managed at an early stage. However, poor patient monitoring can lead to irreversible damage, resulting in the interruption of the treatment, the selection of resistant strains, and finally, in a longer, more complex and more toxic treatment.

## 3. Nanoparticles

### 3.1. Diversity and Versatility of Drug Nanocarriers

Based on the aforementioned observations, there is a clear challenge to optimize TB treatment. The hypothesis of the discovery of new active molecules which would surpass all pre-existing ones is unlikely. Both the scientific and medical communities are well aware that research has been going through a discovery void for the past decades. Indeed, the discovery of new molecules has considerably slowed down during this phase compared to the golden age of antibiotics [52]. Optimizing the potential of current drugs would enable one to overcome many of the previously mentioned constraints. First, targeting the organs of interest would lead to the reduction in the administered doses, as well as in the associated side effects. Then, the development of sustained release systems would allow the reduction in the posology. Finally, such a therapy would improve patient compliance and significantly reduce the risks of relapse. Such is the potential of NPs. In addition, NPs have been widely used for TB diagnosis, but this topic has recently been reviewed [53,54].

**Figure 5:**
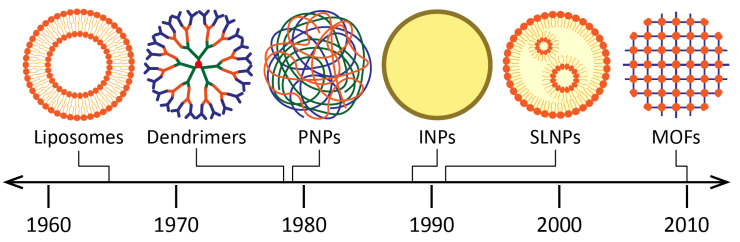
Schematic representation of the main nanosized drug delivery systems for biomedical applications discovered over time. PNPs: polymeric nanoparticles; INPs: inorganic nanoparticles; SLNPs: solid lipid nanoparticles; MOFs: metal–organic frameworks.

Before presenting the usefulness and challenges related to the use of NPs for the treatment of TB, a brief overview of the different types of nanosized DDSs is given. Providing an exhaustive list would be a considerable task beyond our objective. Instead, we give an overview of the main drug nanocarriers. These are chronologically schematized in Figure 5:

- Liposomes were introduced as drug carriers as early as 1965. They are vesicles made of at least one lipid bilayer, which is itself made of phospholipids [55]. The amphiphilic character of these molecules (hydrophilic head, hydrophobic tail) enables the simultaneous encapsulation of active molecules with different solubilities. The applications of liposomes are vast (among others, in the food and cosmetics industries), especially in the biomedical field: the ability of liposomes to encapsulate nucleic acids, enzymes, hormones as well as blood factors, makes them suitable carriers for the treatment of infectious diseases, cancers, gene therapy and for molecular imaging [56]. Other structures such as niosomes [57], phytosomes [58] and transferosomes [59] are also vesicular DDSs of interest.
- Nano(micro)emulsions, first introduced in 1943 [60], are another type of DDS that have gained much attention because of their high loading capacity, ease of preparation, and thermodynamic stability [61,62]. They are defined as a system of water, oil and an amphiphile (surfactant and co-surfactant) which is optically isotropic and thermodynamically stable [63].
- Dendrimers were discovered later, at the end of the 1970s [64]. Their structure consists of a hydrophobic core, with chains of repeating units grafted onto it, branching off each other in a dendritic manner [65]. Functional groups can also be grafted at the periphery. Thus, hydrophilic molecules can be integrated into dendrimers (using the large specific surface conferred by the chains of repeated units) as well as hydrophobic ones (using the core cavity). The major advantages of dendrimers are their homogeneity and small size. Their biomedical applications include infectious diseases, cancers, and gene therapy [66]. INH and RFP, mainly, were incorporated in dendrimers to treat TB [67].
- Inorganic nanoparticles (INPs) are nanocarriers which have also been widely studied for drug delivery [68]. In this group, nanomaterials derived from gold, silica, carbon nanotubes and iron oxides can be found. Gold nanoparticles (AuNPs) have aroused great interest for drug delivery. Indeed, they are chemically inert and non-toxic, and they can be used as contrast agents for medical imaging applications [69]. Unlike other nanocarriers, drugs are usually immobilized on the AuNP surface for their loading, while other ligands and chemical moieties can also be added for their protection and targeting. Silica nanoparticles (SiNPs) are biocompatible nanocarriers that have been used as excipients and food additives for years [70]. They stand out due to their high load capacity, mechanical stability, simplicity of functionalization, and customizable release profiles. Mesoporous SiNPs are of particular interest due to their large surface area. Moreover, iron oxide NPs have also been studied in the medical field, although only imaging applications have reached the market [71]. They have a magnetic behavior which can be useful to guide them (with the help of an external magnetic field) towards the target, thus enhancing the drug release [72].
- Metal–organic frameworks (MOFs) are promising porous nanocarriers which have generated growing interest over the past twenty years. The originality of these structures resides in the combination of metal ions and organic ligands, which assemble to form highly porous networks. This feature enables specific surfaces ranging up to almost 2000 m^2^/g for biocompatible formulations, favorable for drug entrapment [73,74]. The diversity of MOFs is such that nearly one hundred thousand different models are currently deposited in the Cambridge Structural Database. Nanosized MOFs are studied for biomedical applications (infectious diseases and cancers), as well as for industrial uses (gas storage and separation, catalysis, water treatment).
- Solid lipid nanoparticles (SLNPs) were discovered in the early 1990s [75]. They consist of phospholipids whose tails form a solid hydrophobic core surrounded by a surfactant layer. Compared to liposomes, SLNPs offer increased stability for the active molecule (thanks to the solid core), higher encapsulation rates for hydrophobic molecules (since SLNPs do not possess an aqueous core) and the possibility of targeting and sustained release, thanks to the grafting of ligands of interest [76]. In addition, they can be stored for extended periods (up to three years). They are interesting candidates for various administration routes (mainly intravenous and pulmonary), and, as the rest of this review will highlight, they have been widely studied for the treatment of TB.
- Finally, polymeric nanoparticles (PNPs), such as SLNPs, occupy a prominent place in the therapeutic arsenal for the treatment of TB. Polymers are macromolecules formed by repeating covalently linked units (monomers), whose applications for drug delivery have recently been listed in another review [77]. There is a wide variety of polymers, with biodegradable ones being the most widely used: among others, chitosan, PLA (poly(lactic acid)), PLGA (poly(lactic-co-glycolic acid)), and PCL (poly(ε-caprolactone). During the preparation of PNPs, the polymeric chains assemble, often with the help of surfactants for the stabilization of the system. This results in structures suitable for the incorporation of a wide variety of molecules, both hydrophilic and hydrophobic, depending on the properties of the used material. The physicochemical properties of PNPs can be easily modified to design the appropriate nanocarrier for a given pathology, an aspect that will be developed in the following paragraphs.

To conclude this non-exhaustive list, it is important to emphasize that there is no such thing as the ideal drug nanocarrier. Depending on the molecule of interest, each nanocarrier has its own advantages and shortcomings. Common drawbacks include the low encapsulation and loading efficiency of active molecules, uncontrollable (“burst”) release, unsatisfactory reproducibility for industrial production and instability in biological media. Formulation is therefore a crucial step that takes into account both the characteristics of the active molecule and those of its carrier.

Although DDSs have been the subject of extensive research for decades, they still exert modest market influence. Their potential applications in the biomedical field are not limited to infectious diseases, but also include cancers [78] and autoimmune diseases [79]. Regarding TB, a review published in 2005 already stated the advantages of nanotechnologies compared to standard treatments: in particular, high stability for prolonged storage, high levels of encapsulation and loadings of active molecules, incorporation of hydrophilic molecules as well as hydrophobic ones, design for various routes of administration and controlled and the prolonged release of active substances [80]. Thus, over the last years, it has been shown that the various DDSs listed above are also applicable for the treatment of TB [1,2,49,81,82].

### 3.2. Influence of Physicochemical Properties on the Fate of Drug Nanocarriers

As demonstrated above, there is tremendous variety among drug nanocarriers. Indeed, it is possible to play not only with the materials constituting the NPs, but also with the physicochemical properties of the latter, to finely tune the NPs’ fate both in vitro and in vivo. The characteristics of the NPs have a significant impact on their therapeutic efficacy:

- Size plays a major role in the mode of internalization of NPs. It is one of the main parameters studied during their characterization. Thus, NPs of 120 nm–200 nm mainly penetrate inside cells using the clathrin-dependent and caveolin-dependent pathways, while those of more than 200 nm are preferably internalized by macropinocytosis [83]. Those of 250 nm–1 µm are rather taken by phagocytosis. Not all pathways lead to the same intracellular compartments. Indeed, phagocytosis and the clathrin-dependent pathways lead to endosomes, while macropinocytosis leads to lysosomes and the caveolin-dependent pathway leads to caveosomes [84]. Therefore, in the case of TB, adapting the size of NPs so that they target one pathway (and one intracellular compartment) rather than another is a part of the therapeutic strategy itself.
- Depending on the formulation parameters, the shape of NPs can be varied (spheres, cubes, rods and cones), which in turn impact the NP’s intracellular fate [85]. For example, as early as 2006, Chithrani et al. studied the effect of the shape of AuNPs upon their internalization within HeLa cells. They showed that spherical NPs were internalized five times more than rod-shaped ones and hypothesized that this was due to more complex plasma membrane movements for rods than for spheres [86].
- The surface charge is another parameter to consider. Since the plasma membrane is negatively charged, positively charged NPs are more internalized than neutral or negatively charged objects [87]. Moreover, charge can also be used to specifically target an intracellular compartment [85,88]. Indeed, positively charged NPs tend to be internalized by macropinocytosis, while negatively charged NPs rather use the clathrin/caveolin-independent pathway [83], thus leading to different cellular locations.
- Finally, one of the main parameters to consider for the preparation of NPs is surface modification. Indeed, an appropriate surface modification can determine the internalization of NPs within a given cell type, as well as the NP’s fate at the scale of the entire organism [89]. For example, the grafting of amine or carboxyl groups at the surface of nanocarriers gives the latter a more positive or negative charge, respectively, which leads to the consequences explained in the previous paragraph. In addition, the grafting of polyethylene glycol (PEG) at the surface of NPs enables one to prevent the adsorption of opsonins, which makes it possible for NPs to escape the immune system and to prolong their circulation time in the organism.

As a conclusion, the study of the physicochemical properties of NPs is not only a matter of characterization: it is a part of the therapeutic strategy itself. Indeed, a controlled and judicious preparation allows to influence the distribution and internalization kinetics of the NPs and to adjust them according to the pathology to be treated. The question is now is to understand how NPs could be employed to address the problematics linked to the treatment of TB.

## 4. The Potential of Nanoparticles Regarding the Treatment of Tuberculosis

### 4.1. Preamble

**Figure 6:**
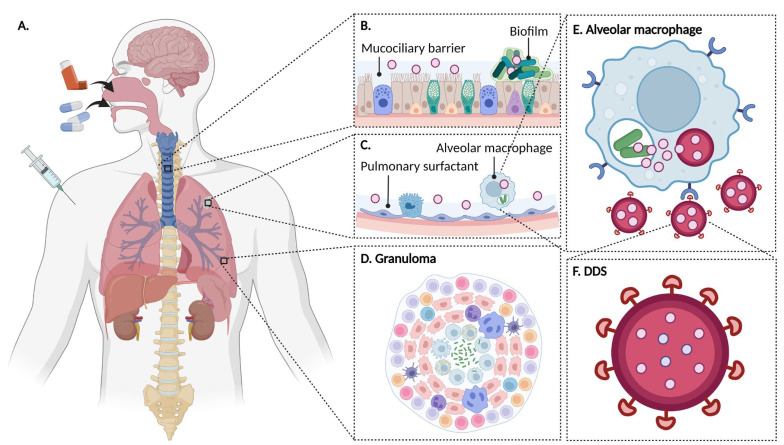
Use of nanosized drug delivery systems (DDSs) to fight tuberculosis. (**A**) Nanosized DDSs can be used to track *Mycobacterium tuberculosis* (*Mtb*) not only in the lungs (the site of infection in most clinical cases) but also in cases of extra-pulmonary tuberculosis, reaching organs such as the bones, brain, eyes, liver, kidneys or spleen. Therefore, DDSs can be administered intravenously, orally or pulmonary, with the latter being of particular interest. (**B**) Trachea and bronchioles constitute the first barrier for the nanosized DDSs during pulmonary administration. Exogenous particles such as NPs can be removed by means of mucociliary clearance, where the periciliary and mucus layers play a crucial role. The *Mtb* biofilm is another obstacle for drug nanocarriers. (**C**) If DDSs overcome the previous barriers, they will later face the clearance mechanisms in the alveoli. In these cavities, DDSs are at the mercy of pulmonary surfactant and the host’s immune defenses. Alveolar macrophages and dendritic cells readily uptake NPs. (**D**) Granulomas are formed in the lungs in order to contain the *Mtb* infection, and are another biological barrier for NPs. (**E**) Targeting alveolar macrophages with NPs is an attractive strategy. Whereas free drugs often poorly penetrate inside infected macrophages, nanocarriers can act as “Trojan horses” to carry drugs inside the cells. Nanocarriers can also be coated with ligands such as mannose for active targeting. (**F**) DDSs are engineered to overcome multiple barriers in the body and achieve drug delivery at *Mtb* locations. Drugs are (co-)encapsulated in the NPs’ cores, whereas coatings can modulate the interactions with the biological medium. Created with BioRender.com.

As previously mentioned, the use of DDSs aims to improve TB treatment. However, as in all diseases, DDSs encounter specific obstacles to their administration. Figure 6A–E illustrate the interest and challenges of using DDSs to treat TB. In the pulmonary route, for example, engineered NPs have to bypass several biological barriers such as trachea and bronchioles [90], mucociliary clearance, granuloma, infected cell membranes, and *Mtb* biofilm [91]. Biofilms are organized structures made up of bacteria adhered to a surface and enclosed by their extracellular matrix, composed of polysaccharides, extracellular polymers, lipids, and DNA. NPs also encounter clearance mechanisms in the alveoli [90]. Thus, DDSs are adequately designed to be able to overcome all these barriers (Figure 6F). The association of drugs with nanocarriers allows their controlled and prolonged release. Additionally, drugs can be co-encapsulated, improving the efficiency of the treatment, and ultimately reducing the doses and side effects. In addition, targeting enables one to increase the bioavailability of drugs. Additionally, as will be mentioned below, DDSs can even potentiate the effect of drugs or act as antimicrobial agents themselves.

**Figure 7:**
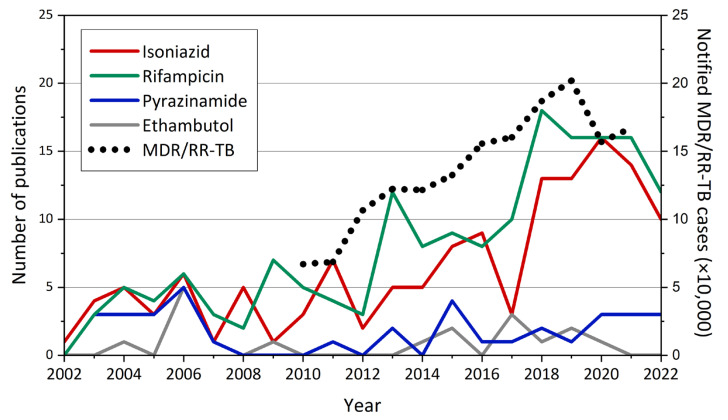
Number of publications discussing the incorporation of first-line antitubercular drugs into nanocarriers. The search was carried out in Scopus in December 2022 using the nanocarriers described in this review as keywords. The dotted line shows the cases of multidrug-resistant/rifampicin-resistant tuberculosis (MDR/RR-TB) according to the annual reports of the World Health Organization. The drop in diagnosed cases from 2019 to 2020 is a consequence of the coronavirus pandemic.

The objective of this review was not to provide an exhaustive list of all nanosized DDSs designed for TB treatment, but rather, through relevant and selected publications, to give an overview of the perspectives offered by NPs. Most studies in the literature deal with the incorporation of the four antibiotics which constitute the first-line treatment: INH, RFP, PZA, and EMB. An interesting fact is worth mentioning: to date, in terms of encapsulation, two of the four drugs (INH and RFP) have generated a much higher number of publications than the other two (PZA and EMB) (Figure 7, Table 1). A possible explanation would be that, since INH and RFP are the mainstays of the first-line treatment, exploiting their potential was considered a priority in the field. Another hypothesis, from a physicochemical point of view, would be that PZA and EMB are more challenging to load in nanocarriers. Interestingly, there is a correspondence between the trends of the number of publications incorporating INH and RFP, and the number of MDR/RR-TB cases (Figure 7). However, to date, only a few studies have dealt with the incorporation of second-line drugs in nanosized DDSs.

The main assets of NPs will be illustrated: (i) delivering antibiotics to the site of infection and increasing their bioavailability; (ii) potentiating the antibacterial effect while reducing the posology; (iii) diminishing the treatment toxicity; (iv) diminishing the treatment complexity; (v) exploiting the antibacterial effect of the nanocarrier itself; and finally, (vi) encapsulating new antitubercular drugs. Other important trends in the field, such as host-directed therapies, will also be presented.

### 4.2. Delivering Antibiotics to the Site of Infection and Increasing Their Bioavailability

In most clinical cases, *Mtb* is located in the lungs. The first-line treatment exclusively relies on oral administration and systemic distribution. The possibility to target the lungs, the main organs of interest, is a great asset for NPs compared to standard treatments [92,93]. The introduction of inhalable formulations would represent unprecedented progress for the treatment of TB. Therefore, it is not surprising that all first-line antitubercular drugs have been studied for pulmonary administration: INH [94,95,96], RFP [97,98,99], PZA [100,101], and EMB [102]. Several routes can be used for drug pulmonary administration [103]. The intranasal, intratracheal and orotracheal routes are effective and commonly used, but also quite invasive, which is why the inhalation route is the most preferred one. Finely designed inhalation devices (pressurized metered-dose inhalers, dry powder inhalers, nebulizers) allow the good penetration of lung tissues and alveoli using dry powders or NP suspensions [90].

**Figure 8:**
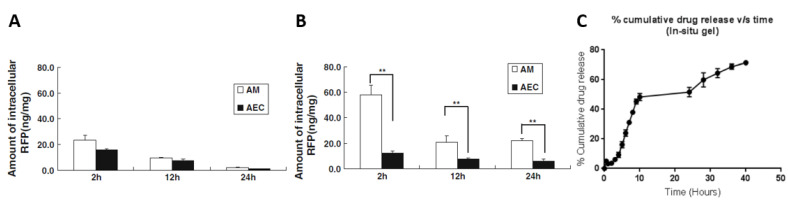
(**A**,**B**) Intracellular rifampicin (RFP) concentrations per mg of protein in alveolar macrophages (AM) (white) and in type II alveolar epithelial cells (AECs) (black) after pulmonary administration of 1 mg/kg in rats. *n* > 3. Symbol ** denotes *p* < 0.05. (**A**) Results for free RFP. (**B**) Results for RFP-loaded solid lipid nanoparticles. Reference [98], copyright (2022) by Copyright Clearance Center, with permission from Springer Nature. (**C**) Percentage of RFP released from chitosan nanoparticles over time in artificial synovial fluid. Reference [104], copyright (2022) by Copyright Clearance Center, with permission from Springer Nature.

In vivo results are generally very promising. In 2013, Chuan et al. developed RFP-loaded SLNPs and tested them via the pulmonary route in rats [98]. The administration of free RFP resulted in concentrations within the alveolar macrophages close to 20 ng/mg of protein 2 h after treatment, barely detectable 24 h later (Figure 8A). Furthermore, the difference in intracellular concentration between alveolar macrophages (therapeutic target) and type II alveolar epithelial cells (surrounding tissue) did not appear to be significant. On the other hand, the use of SLNPs enabled to obtain intracellular RFP concentrations close to 60 ng/mg of protein for alveolar macrophages 2 h after administration (only 10 ng/mg of protein for type II alveolar epithelial cells). These concentrations were maintained at 20 ng/mg of protein 24 h later (Figure 8B). This study demonstrates the ability of NPs to significantly increase the amount of active molecules delivered to the therapeutic target, and to spare surrounding cell types.

However, *Mtb* sometimes escapes from the lungs to reach other organs. In 2021, Prabhu et al. incorporated RFP into chitosan NPs for intra-articular injection to treat osteo-articular TB, one of the most common forms of extra-pulmonary TB [104]. In order to optimize the uptake of NPs, their surface was modified with mannose residues, since the membrane of macrophages bears associated receptors (overexpressed during mycobacterial infections). No improvement regarding the minimum inhibitory concentration (MIC) was detected with NPs compared to free RFP, but the drug release from the NPs appeared to be higher for an acid pH (89% in 12 h for a pH of 5.2) compared to a neutral pH (71% in 12 h for a pH of 7.4). Furthermore, the formulation of NPs in an in situ gelling system enabled to keep them within the intra-articular cavity and to ensure their sustained release (70% in 40 h) (Figure 8C).

In addition, in 2019, Singh et al. designed INH-loaded SLNPs for ocular administration. The complexity of eye tissues renders ocular TB particularly difficult to treat [105]. The tests in rabbits demonstrated not only the absence of toxicity of the NPs regarding the treated tissues, but also an improvement in the bioavailability of INH of 428% compared to free INH. Moreover, with NPs, the antibiotic could be detected in eye tissues up to 24 h after administration (only 12 h if free). Similarly, in 2020, Bazán Henostroza et al. developed RFP-loaded cationic nanoemulsions of approximately 150 nm for the treatment of ocular TB [106]. They were coated with chitosan or polymyxin B. In vitro mucoadhesion tests indicated the establishment of electrostatic interactions between the cationic nanoemulsions and the negatively charged mucin, favorable to improve the residence time in the eye.

**Figure 9:**
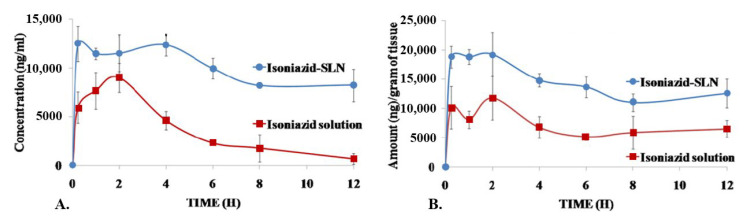
Concentration profiles for free isoniazid (INH) (red) and for INH-loaded solid lipid nanoparticles (SLNPs) (blue) after the oral administration of 25 mg/kg in rats. *n* = 6. (**A**) Results in plasma. (**B**) Results in the brain. Adapted from reference [107], copyright (2022) by Copyright Clearance Center, with permission from Elsevier.

One must also consider the case of miliary TB, where *Mtb* disseminates throughout the body and the systemic distribution of active molecules then becomes a therapeutic objective. In 2013, Bhandari et al. proposed to administer INH-loaded SLNPs orally [107]. The formulation enabled to reach concentrations higher than those obtained with free INH in the plasma and in the brain, thanks to the small size of the NPs (less than 50 nm), which allowed to avoid the first-pass hepatic metabolism and to prolong the circulation time in the organism (Figure 9).

Extra-pulmonary forms of TB in the brain are difficult to treat, owing to problems associated with drug delivery across the blood–brain barrier. Baijnath et al. showed, in a model of *Mtb*-infected mice, that a treatment with LZN and CFZ allowed preventing the development of extra-pulmonary TB in the central nervous system [108]. De Castro et al. developed CFZ-loaded PLGA-PEG NPs functionalized with a transferrin receptor-binding peptide to target the brain for the treatment of central nervous system TB [109]. In vitro studies with brain endothelial hCMEC/D3 cells showed that encapsulation significantly reduced drug cytotoxicity. In addition, the functionalized NPs exhibited better cellular interactions and higher CFZ permeability through the endothelial cell monolayers as compared to non-functionalized ones.

Lymphatic TB is a common type of extrapulmonary TB, which affects the peripheral lymph nodes and requires a long-term treatment comprising multiple antibiotics, resulting in an increased rate of MDR-TB. To deal with this issue, water-in-oil nanoemulsion was prepared using sunflower oil, Span 80 and Tween 80, incorporating LZN [110]. In vivo studies in rats confirmed the lymphatic targeting with a high amount of drug accumulated at the targeted organ after 8 h. Hussain et al. also used nanoemulsions to treat systemic and cutaneous TB [111]. RFP was successfully loaded in cationic nanoemulsions prepared using capmul, labrasol and acconon, which exert anti-*Mycobacterium* effects. This strategy enhanced the drug permeation across the skin, increased the therapeutic efficacy and reduced dose-related side effects.

The examples cited here prove that NPs can be successfully used to deliver antibiotics to one or more organs of interest, depending on the location of *Mtb*. To our knowledge, no nanoformulations have been studied for the treatment of less common types of TB (pleural, gastrointestinal or urogenital).

As mentioned above, it is also possible to modify the surface of NPs with various ligands, including mannose residues, to favor their internalization by macrophages in infected tissues [96,104,112,113,114]. As an additional example, Marcianes et al. (2020) developed biodegradable PLGA microparticles for the pulmonary administration of gatifloxacin, using labrafil as a surface modifier to actively target alveolar macrophages [115]. It is noteworthy that, at the intracellular level, the acid pH within the lysosomes induces a better release of the active molecules [96,104,112,116,117]. We further show that NPs enable the reduction in administered doses for a similar, if not more important, antibacterial effect.

### 4.3. Potentiating the Antibacterial Effect While Reducing the Posology

**Figure 10:**
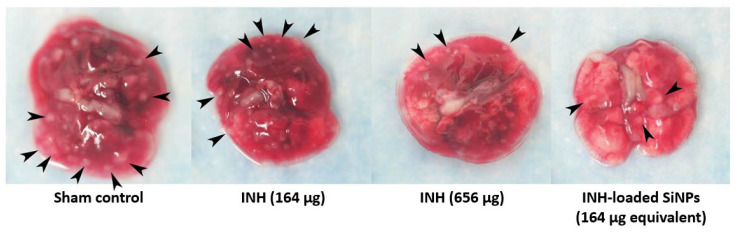
Lung tissues from untreated mice, from mice treated with free isoniazid (INH), and from mice treated with INH-loaded mesoporous silica nanoparticles (SiNPs) administered intravenously or subcutaneously. Black arrows indicate granulomas visible to the naked eye. Adapted from reference [118], copyright (2022) by Copyright Clearance Center, with permission from John Wiley and Sons.

Hwang et al. investigated in 2015 INH-loaded mesoporous SiNPs [118]. Tests were carried out in vivo on mice infected with *Mtb* and treated with NPs, using the intravenous or subcutaneous route. At equivalent doses, NPs produced an antibacterial effect four times more important than free INH, in the lungs (reduction in the bacterial burden of 1.3 log_10_ colony-forming units (CFUs)), in the liver (reduction of 2.1 log_10_ CFUs), and in the spleen (reduction of 3.9 log_10_ CFUs). The effectiveness of the formulation could be observed even with the naked eye on the lung tissues extracted from the treated mice, which showed a reduced number of granulomas compared to those of mice treated with free INH (Figure 10).

Combination therapy has been demonstrated to be a potentially effective treatment for MDR-TB [119]. MOX, econazole and ETH were incorporated into PLGA NPs to treat MDR-TB-infected mice. Whereas eight weeks of oral administration of individual NPs for each drug showed a limited reduction in CFUs in the lungs and in the spleen, only eight doses of a combination of the three nanoformulations successfully reduced the CFUs in both the lungs and the spleen.

Other studies demonstrated the ability of NPs to potentiate the effects of antibiotics. In 2011, Saraogi et al. synthesized mannosylated INH-loaded gelatin NPs for intravenous administration [113]. Tests were carried out in vivo on *Mtb*-infected mice. Free INH allowed a reduction in the bacterial burden from 6.1 log_10_ CFUs to 4.83 log_10_ CFUs in the lungs and from 3.0 log_10_ CFUs to 2.54 log_10_ CFUs in the spleen. However, with INH-loaded NPs, this effect was multiplied by a factor of nearly 2.5 (*p* ≤ 0.001), since, at equivalent doses, the bacterial burden dropped to 2.13 log_10_ CFUs in the lungs and to 1.08 log_10_ CFUs in the spleen.

Furthermore, it is interesting to note that NPs loaded with antitubercular drugs are able to exert an antibacterial effect on other pathogens, such as *Staphylococcus aureus*, *Pseudomonas aeruginosa*, *Mycobacterium avium* [94], *Escherichia coli* [120] and *Mycobacterium smegmatis* [96]. Thus, NPs are able to potentiate the effects of active molecules at equivalent or reduced doses.

### 4.4. Maximizing Patient Compliance by Reducing Treatment Toxicity

Vectorizing an antibiotic enables one to reduce the administered doses, and therefore, to reduce its toxicity. INH is a drug with side effects, such as hepatotoxicity. Saraogi et al. investigated the hepatoprotective effect of gelatin NPs by studying several serum markers (bilirubin, SGPT (serum glutamate pyruvate transaminase) and ALP (alkaline phosphatase)) to assess liver damage [113]. These markers were quantified two days after the administration of the last dose of INH (free or encapsulated into NPs), after one month of treatment. The assays (*n* = 3) revealed that:

- For the untreated mice, the concentrations of total bilirubin, SGPT and ALP, were, respectively, 0.24 mg/100 mL, 32.2 IU/L and 33.3 IU/L.
- For the mice treated with free INH, these concentrations were, respectively, 0.63 mg/100 mL, 57.5 IU/L and 47.6 IU/L.
- For the mice treated with INH-loaded NPs, these concentrations were, respectively, 0.23 mg/100 mL, 33.45 IU/L and 32.61 IU/L.

A significant increase in serum marker levels was observed with free INH, while no difference was observed with INH-loaded NPs compared to the untreated condition (*p* ≤ 0.05). It was concluded that the nanoformulation allowed not only a superior antibacterial effect for equivalent doses of INH compared to the standard treatment, but also a reduction in the side effects of INH.

**Figure 11:**
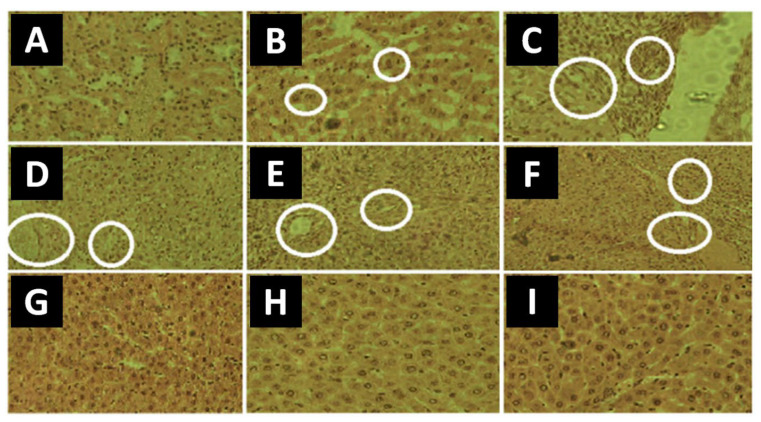
Histological sections of livers from untreated rats, from rats treated with free rifampicin (RFP) and from rats treated with RFP-loaded chitosan nanoparticles (NPs) (magnification: ×400). (**A**) Untreated group. (**B**–**G**) Group treated with free RFP (regions of interest circled in white). (**B**) Hydropic degeneration. (**C**) Lobular hepatitis. (**D**) Apoptosis. (**E**) Vein congestion. (**F**) Portal tract expansion. (**G**) Group treated with free RFP. (**H**) Group treated with empty chitosan NPs. (**I**) Group treated with RFP-loaded chitosan NPs. Adapted from reference [114], used under Creative Commons BY 4.0 license.

Similar findings were reported by Rauf et al. in the case of RFP-loaded chitosan NPs [114]. After oral treatment, rats were kept under observation for two weeks before being sacrificed, so that the blood, serum and organs could be analyzed. The assays (*n* = 5) revealed that the group treated with free RFP showed significantly higher levels of transaminases compared to the untreated group, as well as significantly lighter livers (which indicated liver damage). On the other hand, the parameters associated with the group treated with RFP-loaded NPs were similar to those associated with the untreated group, which demonstrated the hepatoprotective effect of the NPs. This effect could even be observed visually by studying the histological sections of livers from each group (Figure 11). While no notable difference was observed between the untreated group and the group treated with NPs, the group treated with free RFP showed considerable liver damage (swelling, binucleation, degeneration, apoptosis and necrosis).

Like Saraogi et al. [113], Rauf et al. [114] demonstrated that the vectorization of antibiotics enables to potentiate their antibacterial effects: in rabbits, the NPs of Rauf et al. increased the bioavailability of RFP by a factor of almost 16, as well as reduced the side effects of RFP.

### 4.5. Maximizing Patient Compliance by Reducing Treatment Complexity

Minimizing the toxicity of the treatment is an essential requirement to ensure patient compliance. The potential of NPs goes even further. As previously explained, the current treatment of TB relies on the daily intake of several drugs for months, a posology which is particularly difficult to respect in emerging countries. However, a few studies report formulations enabling the co-encapsulation of several antibiotics, which consequently leads to a considerable reduction in the number of administered doses. In 2020, Khatak et al. managed to co-encapsulate INH, RFP and PZA within SLNPs and performed in vitro tests against *Mycobacterium marinum* [121]. In 2021, Changsan et al. co-encapsulated INH and PZA within chitosan NPs designed for pulmonary delivery [122].

In previous studies, in 2005, Zahoor et al. developed nebulizable alginate NPs loaded with INH, RFP and PZA [123]. Free antibiotics were detectable in plasma for up to 12 h to 24 h after administration in *Mtb*-infected guinea pigs. When formulated within NPs, INH, RFP and PZA were detectable in plasma for up to 14 days, 10 days and 14 days, respectively. All the pharmacokinetic parameters (maximum plasma concentration (C_max_), time to reach the maximum plasma concentration (T_max_) and area under the curve (AUC)) were significantly higher when the animals were treated with NPs (*p* < 0.001). In addition, the three antibiotics were detected at concentrations above the MIC in the lungs, in the liver and in the spleen for up to 15 days after administration.

These results illustrate the potential of NPs regarding the sustained release of active molecules. The same research group further tested the therapeutic effect of their formulation while modifying the posology of the treatment. One group of animals served as a control, another received the free antibiotics (one administration per day, i.e., 45 doses), another received empty NPs (one administration every 15 days, i.e., 3 doses), and another received NPs loaded with antibiotics (one administration every 15 days, i.e., 3 doses) (*n* = 5–6). At the end of the treatment, the bacterial burden was assessed in the lungs and in the spleen. For untreated animals and for those treated with empty NPs, the load was 5.8 log_10_ CFUs in the lungs and 5.9 log_10_ CFUs in the spleen. However, for animals treated with free antibiotics (45 doses) and for those treated with NPs loaded with antibiotics (3 doses), *Mtb* was undetectable, both in the lungs and in the spleen.

Thus, Zahoor et al. proposed a therapeutic regimen which is as effective as the standard treatment, while reducing the number of doses by a factor of 15. Similarly, in 2005, Pandey et al. formulated SLNPs loaded with INH, RFP and PZA for oral administration in *Mtb*-infected [124]. While free antibiotics were detected in plasma for up to 12 h after administration, the detection period extended up to 8 days for NPs. Moreover, 5 doses of NPs produced a therapeutic effect similar to that of the standard treatment (46 doses, for an undetectable bacterial load within the studied organs). In 2006, the same research group proposed PLGA NPs containing INH, RFP and PZA, or only EMB. They were administered orally in infected mice, in an attempt to reach therapeutic concentrations in the brain [125]. The vectorization of antibiotics enabled to increase the drugs’ bioavailability by a factor varying from 15 to 30. Furthermore, 10 doses of NPs produced a therapeutic effect similar to 46 doses of free antibiotics, eventually leading to undetectable bacterial loads in the brain.

Finally, with the scope of treating MDR-TB, Abdelghany et al. co-entrapped amikacin and MOX into alginate-modified PLGA NPs and assessed the anti-mycobacterial activity in *Mtb*-infected macrophages [126]. The bacterial viability was reduced to only 0.6% as compared to the untreated group, and the results were better than in the case of a formulation with just one drug.

### 4.6. Exploiting the Antibacterial Effect of the Nanocarrier Itself

**Figure 12:**
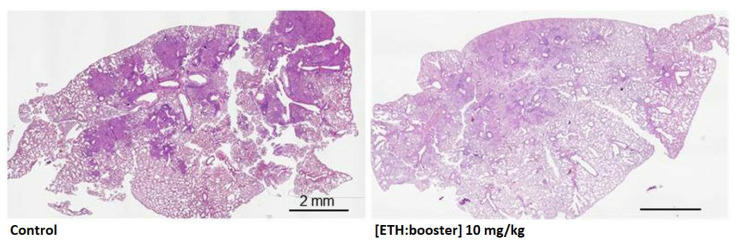
Histological sections of lungs from *Mycobacterium tuberculosis*-infected mice, untreated (**left**) or treated with β-cyclodextrin-based nanoparticles loaded with ethambutol (ETH) and BDM41906 (booster) 6 times in 2 weeks by the endotracheal route (**right**). Adapted from reference [127], used under Creative Commons BY 4.0 license.

It is noteworthy that a recent co-encapsulation study demonstrated that NPs cannot only efficiently load drugs, but also actively play a role in fighting the infection [127]. β-cyclodextrin (CD)-based NPs act as molecular sponges, soaking drugs from aqueous solutions. The second-line antitubercular drug ETH was co-encapsulated with one of its boosters (BDM41906, which inhibits an enzyme (EthR), which itself inhibits ETH). The antibacterial effect of this formulation was assessed in vivo in mice infected with *Mtb* and treated by the endotracheal route 6 times in 2 weeks. Figure 12 shows the comparison of two histological sections: one carried out on the lung of an untreated infected mouse, the other carried out on the lung of a treated infected mouse. The first section shows lung tissue heavily damaged by *Mtb* infection, while the second one shows practically no damaged tissue. This demonstrates the therapeutic efficacy of the CD-based formulation.

For the benefit of the patient, the “ideal” formulation would be that in which each of its components plays a role in fighting the disease. With a formulation devoid of “inert” components, the needed administered doses would be reduced. Two approaches were followed in the case of the ETH–booster pair.

Firstly, ETH and its booster BDM43266 were chemically linked to produce a co-drug [128]. Remarkably, the co-drug was capable of self-assembling into NPs of approximately 200 nm. Taking into account the linker between the two active molecules, the global drug loading could be estimated at almost 80%, which was a significant gain as compared to the CD-based NPs (loading of 10%) [127].

**Figure 13:**
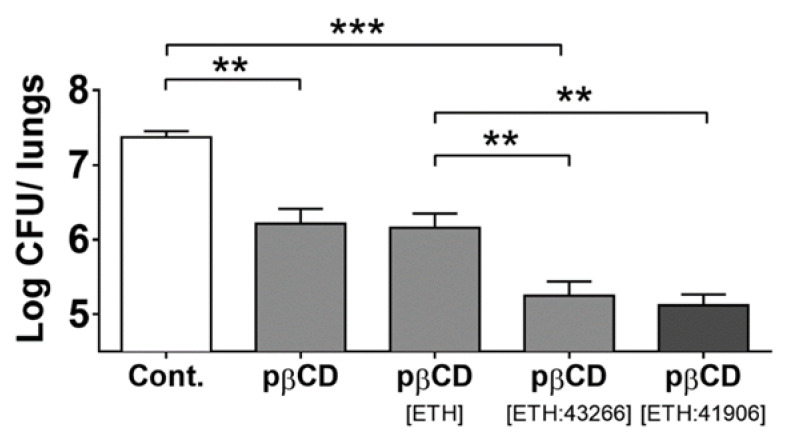
Effect of the administration of β-cyclodextrin-based nanoparticles (pβCD) loaded with ethionamide (ETH) on the bacterial load in the lungs of *Mycobacterium tuberculosis*-infected mice. The different conditions (control group, empty pβCD, ETH-loaded pβCD and ETH- and booster-loaded pβCD (BDM43266 or BDM41906)) are compared. Bacterial count was performed after the endotracheal administration of 6 doses of 7.5 mg of nanoparticles. Symbols ** and *** denote *p* < 0.01 and *p* < 0.001, respectively. Reference [131], used under the standard ACS Author’s Choice/Editor’s Choice Usage Agreement.

Secondly, the nanocarrier itself can have an antibacterial effect. It was discovered that CD-based NPs [127,129,130] have intrinsic antibacterial properties [131]. The antibacterial effect of different formulations (β-CD-based NPs, empty, loaded with ETH, or loaded with ETH and a booster (BDM43266 or BDM41906)) was studied in vivo in mice infected with *Mtb* and treated by the endotracheal route. It was found that empty NPs enabled a reduction in the bacterial load in the lungs of 1 log CFUs compared to the untreated group (Figure 13). It is noteworthy that, when ETH was encapsulated with one of its boosters, the bacterial load in the lungs was decreased by another 1 log CFUs.

In addition to their ability to potentiate the effect of ETH and its boosters at low doses, the study showed that the NPs themselves acted according to a double mechanism. On the one hand, by inducing the depletion of cholesterol from the plasma membrane of macrophages, the NPs prevented *Mtb* from reaching its intracellular niche. On the other hand, the NPs induced the apoptosis of alveolar macrophages within the lungs, thus depriving *Mtb* of its sanctuary.

CD-based NPs are among rare organic NPs endowed with intrinsic antibacterial properties. Mainly, INPs (metallic ones, notably) are well known for their antibacterial properties. For instance, INH-loaded selenium NPs favor *Mtb* localization within lysosomes [112]. Similarly, RFP-loaded ZnO NPs cause irreversible damage to the membrane of *Mycobacterium smegmatis* (a surrogate of *Mtb*) and are also effective against resistant strains of *Mycobacterium bovis* [132].

**Table 1:** Overview of the publications dedicated to the encapsulation of first-line antitubercular drugs, ranked by active molecule and by chronological order. PdI: polydispersity index; drug encapsulation: quantity of encapsulated molecule compared to the quantity of molecule used during the preparation of nanovectors, expressed as a percentage; drug loading: mass of encapsulated molecule compared to the total mass of nanovectors, expressed as a percentage.

Physicochemical Properties		Biological Data
Isoniazid (INH)		
**Chitosan–tripolyphosphate NPs** [94]		
Preparation: ionic gelation Size: 249 nm and 449 nm PdI: For 249 nm NPs: 0.191 For 449 nm: 0.240 ζ potential: For 249 nm NPs: 37.7 mV For 449 nm NPs: 38.9 mV Drug encapsulation: For 249 nm NPs: 13% For 449 nm NPs: 17% Drug loading: For 249 nm NPs: 4% For 449 nm NPs: 6%	In vitro	Drug release: For 249 nm NPs: 50% in 4 h, 95% in 6 days For 449 nm NPs: 40% in 4 h, 80% in 6 days Effect against *Staphylococcus aureus* and *Pseudomonas aeruginosa*: Empty NPs: 8-fold reduction in the MIC compared to free INH INH-loaded NPs: 64-fold reduction in the MIC compared to free INH Effect against *Mycobacterium avium*: Empty NPs: no reduction in the MIC compared to free INH INH-loaded NPs: 16-fold reduction in the MIC compared to free INH
	In vivo	-
**Gelatin NPs** [113]		
Preparation: two-step desolvation Mannose-conjugated NPs: Size: 387 nm PdI: 0.262 ζ potential: 10.21 mV Drug encapsulation: 43%	In vitro	Drug release, for a pH of 7.4: INH-loaded NPs: 40% in 4 h, 92% in 120 h Maximum cell uptake in 6 h for J774 cells
	In vivo	Animal model: mouse INH-loaded NPs: 4-fold higher concentration in the plasma, 9-fold higher concentration in the lungs, and 10-fold lower concentration in the kidney compared to free INH Sustained release compared to free INH Effect against *Mtb*: INH-loaded NPs: 2.5-fold reduction in CFUs in the lung and in the spleen compared to free INH No hepatotoxicity
**SLNPs** [107]		
Preparation: microemulsification Size: 48 nm PdI: 0.266 ζ potential: −0.101 mV Drug encapsulation: 69%	In vitro	Drug release, for a pH of 6.8: Free INH: 100% in 5 h INH-loaded NPs: 65% in 24 h
	In vivo	Animal model: rat INH-loaded NPs: significantly higher concentrations in the plasma and in the brain, but not in the liver and in the kidney, compared to free INH
**PLGA-PEG-PLGA NPs** [133]		
Preparation: double emulsification Size: 250 nm to 400 nm Drug encapsulation: 13%–19% Drug loading: 6%–9%	In vitro	INH-loaded NPs: initial burst release followed by sustained release compared to free INH
	In vivo	Animal model: rat INH-loaded NPs: sustained release and 28-fold higher bioavailability compared to free INH
**Mesoporous SiNPs** [118]		
Preparation: formation of liquid-crystalline mesophases of surfactant, in situ polymerization of orthosilicic acid Size: 50 nm and 100 nm Drug loading:50 nm NPs: 3% 100 nm NPs: 6%	In vitro	Effect against *Mtb*: INH-loaded NPs: similar antibacterial effect compared to free INH
	In vivo	Animal model: mouse Effect against *Mtb*: 50 nm NPs: 2-fold higher antibacterial effect compared to free INH 100 nm NPs: 4-fold higher antibacterial effect compared to free INH
**Selenium NPs** [112]		
Preparation: sodium selenite reduction and chitosan stabilization Mannose-conjugated NPs: Size: 45 nm	In vitro	Drug release: For a pH of 7.4: 45% in 48 h For a pH of 5.3: 80% in 48 h Effect against *Mtb*: Empty NPs: intrinsic antibacterial effect INH-loaded NPs: synergistic antibacterial effect against intracellular bacteria Promotion of *Mtb* localization into lysosomes No toxicity towards THP-1 cells
	In vivo	-
**SLNPs** [105]		
Preparation: microemulsification Size: 149 nm PdI: 0.15 ζ potential: −0.35 mV Drug encapsulation: 65% Drug loading: 40%	In vitro	Drug release, for a pH of 7.2: Free INH: 100% in 7 h INH-loaded NPs: 28% in 4 h, 45% in 6 h, 94% in 48 h Ex vivo corneal permeation: INH-loaded NPs: 2.5-fold higher compared to free INH Effect against *Mtb*: INH-loaded NPs: 7.1-fold reduction in the MIC compared to free INH
	In vivo	Animal model: rat and rabbit INH-loaded NPs: 428% higher bioavailability compared to free INH Drug release:Free INH: detection for up to 12 h INH-loaded NPs: detection for up to 24 h No ocular toxicity
**Magnetic NPs** [120]		
Preparation: coprecipitation Lipoaminoacid-modified NPs: Size: 13 nm ζ potential: −19.8 mV Drug loading: 3%	In vitro	Effect against *Staphylococcus aureus*, *Escherichia coli* and *Pseudomonas aeruginosa*: Free INH: MIC90 of >500 µg/mL INH-loaded NPs: MIC90 of 38 µg/mL Effect against *Mtb*: Free INH: MIC90 of 1.26 µg/mL INH-loaded NPs: MIC90 of 1.08 µg/mL
	In vivo	-
**MIL-100 MOFs in mannitol microspheres** [95]		
Preparation: spray-drying Size: 137 nm ζ potential: −18 mV Drug loading: 30%	In vitro	Drug release, for a pH of 7.4: In milli-Q water: 21% in 0 h, 27% in 48 h In PBS: 44% in 0 h, 84% in 120 h No toxicity towards A549 cells
	In vivo	-
**SLNPs** [134]		
Preparation: ultrasonication of crude emulsion Mannose-conjugated NPs: Size: 236 nm PdI: 0.24 ζ potential: −19 mV Drug encapsulation: 75% Drug loading: 10%	In vitro	Drug release: For a pH of 7.4: 59% in 9 h For a pH of 5.5: 83% in 9 h Mannose-conjugated NPs: higher cell uptake in macrophages (97%) compared to non-modified NPs (42%) No toxicity towards RAW264.7 cells and A549 cells
	In vivo	Animal model: rat Effect against *Mycobacterium smegmatis*: Empty NPs: decrease in CFUs of 60% INH-loaded NPs: decrease in CFUs of 83%
**Rifampicin (RFP)**		
**PLA microspheres** [116]		
Preparation: modified solvent evaporation Size: 800 nm to 8 µm Drug loading: 19%	In vitro	Drug release: For a pH of 9.8: 10% in 14 h For a pH of 7.4: 20% in 14 h For a pH of 3.0: 55% in 14 h
	In vivo	-
**PLGA NPs in porous NP-aggregate particles** [97]		
Preparation: solvent evaporation and spray-drying Size: 195 nm PdI: 0.06 ζ potential: −33 mV Drug loading: 14%	In vitro	RFP-loaded NPs: burst release (80%) followed by slower release for 8 h
	In vivo	Animal model: guinea pig Free RFP: low or no levels in the lungs 8 h post-treatment RFP-loaded NPs: higher levels in the lungs 8 h post-treatment
**SLNPs** [98]		
Preparation: modified lipid film hydration Size: 830 nm	In vitro	RFP-loaded NPs: significantly higher intracellular amounts in alveolar macrophages than in alveolar epithelial type II cells compared to free RFP No toxicity towards A549 and NR8383 cells
	In vivo	Animal model: rat RFP-loaded NPs: significantly higher intracellular amounts in alveolar macrophages than in alveolar epithelial type II cells compared to free RFP Significantly higher intracellular concentrations (and for a longer time) in alveolar macrophages compared to free RFP
**Chitosan NPs** [117]		
Preparation: modified emulsion ionic gelation Size: 222 nm Drug encapsulation: 44% Drug loading: 43%	In vitro	Drug release: For a pH of 7.4: 5% to 8% in 1 h For a pH of 6.8 or 5.2: 8% to 13% in 1 h After 1 h, constant drug release, up to 90% in the range of 28 h–34 h
	In vivo	-
**PLGA-lipid hybrid microparticles** [135]		
Preparation: spray-drying Hybrid system of lipid NPs encapsulated within a PLGA NP matrix: Size: 110 nm PdI: 0.15 ζ potential: −7.12 mV Drug encapsulation: 100% Drug loading: 12%	In vitro	Drug release, for a pH of 7.4: RFP-loaded NPs: 8% in 1 h in simulated lung fluid (protection of the drug before phagocytosis), 41% in 48 h in artificial lysosomal fluid Effect against intracellular *Staphylococcus aureus*: Free RFP: no reduction in CFUs until 5 µg/mL RFP-loaded NPs: 4-fold reduction in CFUs for 0.5 µg/mL
	In vivo	-
**ZnO NPs** [132]		
Preparation: precipitation in liquid media Size: 11 nm ζ potential: 19.1 mV	In vitro	Effect against *Mycobacterium smegmatis*: Free RFP: reduction in CFUs, but not after 36 h Empty NPs: no reduction in CFUs up to 60 h RFP-loaded NPs: significant reduction in CFUs compared to free RFP, up to 60 h; irreversible bacterial membrane damage
	In vivo	-
**Alginate-chitosan NPs** [99]		
Preparation: ionic gelation Encapsulation of RFP and ascorbic acid: RFP: Drug encapsulation: 50% Drug loading: 24% Ascorbic acid: Drug encapsulation: 16% Drug loading: 38%	In vitro	Effect against *Staphylococcus aureus*: Free RFP: MIC of 0.2 µg/mL RFP-loaded NPs: MIC of <0.025 µg/mL Effect against methicillin-resistant *Staphylococcus aureus*: Free RFP: 3.125 µg/mL RFP-loaded NPs: 1.6 µg/mL Effect against *Mtb*: Free RFP: MIC of 0.78 µg/mL–1.25 µg/mL RFP-loaded NPs: MIC of 0.039 µg/mL –0.31 µg/mL
	In vivo	Animal model: rat Intratracheal administration, efficient penetration of the airway mucus, distribution throughout the lung tissues
**Chitosan NPs** [104]		
Preparation: ionic gelation Mannose-conjugated NPs: Size: 142 nm PdI: 0.154 ζ potential: 38.5 mV Drug encapsulation: 71% Non-conjugated NPs: Size: 138 nm PdI: 0.173 ζ potential: 42.6 mV Drug encapsulation: 74%	In vitro	Mannose-conjugated NPs: Drug release: For a pH of 7.4: 71% in 12 h For a pH of 5.2: 89% in 12 h Incorporation in in situ gelling system: 70% in 40 h Effect against *Mtb*: RFP-loaded NPs: MIC of 0.009 µg/mL
	In vivo	-
**Chitosan NPs** [114]		
Preparation: ionic gelation Mannose-conjugated NPs: Size: 300 nm ζ potential: 18 mV Drug encapsulation: 73% Drug loading: 40%	In vitro	-
	In vivo	Animal model: rat and rabbit RFP-loaded NPs: 19-fold higher permeation across everted rat intestines compared to free RFP 16-fold higher oral bioavailability in rabbits compared to free RFP Hepatoprotective effect
**SLNPs** [136]		
Preparation: hot ultrasonication Chitosan-coated NPs: Size: 524 nm ζ potential: 30 mV Drug encapsulation: 90% Drug loading: 5% Non-coated NPs: Size: 245 nm ζ potential: −30 mV Drug encapsulation: 89% Drug loading: 5%	In vitro	Drug release: Chitosan-coated NPs: For a pH of 7.4: 34% in 8 h For a pH of 4.5: 25% in 8 h Non-coated NPs: For a pH of 7.4: 50% in 8 h For a pH of 4.5: 50% in 8 h No toxicity towards A549 cells
	In vivo	-
**Pyrazinamide (PZA)**		
**PLGA NPs** [100]		
Preparation: double emulsion-solvent evaporation Size: 173 nm PdI: 0.05 ζ potential: −1 mV Drug encapsulation: 8% Drug loading: 3%	In vitro	-
	In vivo	-
**Eudragit RS-100 NPs** [101]		
Preparation: double emulsion-solvent evaporation Size: 46 nm–300 nm PdI: 0.237–0.823 ζ potential: 3.23 mV–25.2 mV Drug encapsulation: 61%–81% Drug loading: 13%–43%	In vitro	Drug release, for a pH of 6.8: Free PZA: 90% in 6 h, no further release PZA-loaded NPs: rapid release phase up to 11 h, slower release phase over 24 h (approximately 80%) Important uptake in alveolar macrophages 2 h after administration
	In vivo	-
**Ethambutol (EMB)**		
**Graphene oxide with iron oxide magnetite NPs** [137]		
Size: 9 nm Drug loading: 34%	In vitro	Drug release, for a pH of 7.4 or 4.8: Free EMB: 100% in 10 min EMB-loaded NPs: 100% in 50 h Effect against *Mycobacterium smegmatis*: EMB-loaded NPs: MIC of 6.25 µg/mL No toxicity towards 3T3 cells
	In vivo	-
**PCL NPs** [138]		
Preparation: double emulsification Size: 270 nm	In vitro	Effect against BCG: J774A.1 cells, free EMB or EMB-loaded NPs: decrease in percentage of infected cells from 85% to 30%
	In vivo	Animal model: mouse Effect against BCG: 18% of EMB-loaded NPs taken up by the lungs
**SLNPs** [102]		
Preparation: hot homogenization and ultrasonication Size: 58 nm PdI: 0.253 Drug encapsulation: 99% Drug loading: 30%	In vitro	Drug release: Free EMB: 47% in 8 h EMB-loaded NPs: 34% in 8 h No toxicity towards A549 cells compared to free EMB
	In vivo	-
**Combinations**		
**SLNPs: INH + RFP + PZA** [124]		
Preparation: emulsion-solvent diffusion Drug encapsulation: INH: 45% RFP: 51% PZA: 41%	In vitro	-
	In vivo	Animal model: mouse Bioavailability: Free drugs: detection in the plasma for up to 12 h Loaded NPs: detection in the plasma for up to 8 days Effect against *Mtb*: Free drugs: 46 doses Loaded NPs: 5 doses In both cases, undetectable CFUs in the lungs and in the spleen
**Alginate NPs: INH + RFP + PZA** [123]		
Preparation: cation-induced gelification Size: 236 nm PdI: 0.439 Drug encapsulation: INH: 70% to 90% RFP: 80% to 90% PZA: 70% to 90%	In vitro	-
	In vivo	Animal model: guinea pig Bioavailability: Free drugs: detection in the plasma for up to 14 h Loaded NPs: detection in the plasma for up to 14 days Effect against *Mtb*: Free drugs: 45 doses Loaded NPs: 3 doses In both cases, undetectable CFUs in the lungs and in the spleen
**PLGA NPs: INH + RFP + PZA; EMB** [125]		
Preparation: emulsion-solvent evaporation EMB encapsulated separately: drug encapsulation: INH: 67% RFP: 56% PZA: 69% EMB: 43%	In vitro	-
	In vivo	Animal model: mouse Bioavailability: Free drugs: detection in the plasma for up to 12 h; detection in the brain for up to 1 day, except for EMB (6 days) Loaded NPs: detection in the plasma for up to 8 days for INH and PZA, 5 days for RFP, and 3 days for EMB; from 15- to 30-fold higher bioavailability; detection in the brain for up to 9 days Effect against *Mtb*: Free drugs: 46 doses Loaded NPs: 10 doses In both cases, undetectable CFUs in the brain
**SLNPs: INH + RFP + PZA** [121]		
Preparation: microemulsion Size: 188 nm PdI: 0.568 ζ potential: −47.4 mV Drug encapsulation: INH: 84% RFP: 86% PZA: 81%	In vitro	Drug release: Free drugs: For a pH of 6.8: INH: 95% in 1 h RFP: 92% in 1 h PZA: 96% in 1 h For a pH of 1.2: INH: 92% in 1 h RFP: 87% in 1 h PZA: 89% in 1 h Loaded NPs: For a pH of 6.8: INH: 6% in 1 h RFP: 12% in 1 h PZA: 10% in 1 h For a pH of 1.2: INH: 8% in 1 h RFP: 9% in 1 h PZA: 10% in 1 h Effect against *Mycobacterium marinum*: Loaded NPs: 2-fold reduction in bacterial load compared to free drugs
	In vivo	-
**Chitosan NPs: INH + PZA** [122]		
Preparation: ionic gelation Size: 250 nm–576 nm PdI: 0.3–0.4 ζ potential: 25.92 mV–37.44 mV Drug encapsulation: INH: 25%–30% PZA: 25%–30%	In vitro	No toxicity towards NCI-H358, A549 and NR8383 cells Low levels of IL-1β, TNF-α and NO after administration
	In vivo	-

### 4.7. Encapsulating New Antitubercular Drugs

Although the large majority of studies deal with the encapsulation of first-line drugs, more recently, new drugs such as LZN, BDQ, DLM and PMD have also been encapsulated (Table 2). In 2018, Jary et al. studied the encapsulation of BDQ using lipid NPs [139]. The cytotoxicity of the NPs in human cells was detected above 1 mg/mL, a value much higher than the concentration necessary to reach the MIC (between 1 µg/mL and 2 μg/mL). Surprisingly, the fate of the NPs in mice was similar regardless of whether they were coated with trimannose, in an attempt to target macrophages. Thus, this study leaves compelling questions about the strategy of targeting the mannose receptors on macrophages. The same research group carried out a similar study, but comparing lipid NPs with chitosan nanocapsules, both commonly proposed for different administration routes [140]. Although coating the nanocapsules with PEG (stabilizing agent) improved their stability, it also promoted drug release. In in vitro studies, it was shown that the maximum tolerable concentrations were much higher than those needed to achieve the same effectiveness as with the free drug.

In a recent study, Huck et al. investigated the aerosol deposition of dry-powder microparticles of spray-dried BDQ-loaded liposomes [141]. The liposomes were functionalized with fucose to target THP-1 cells, peripheral blood monocyte- and lung tissue-derived macrophages. However, the presence of pulmonary surfactant masked the effect of active targeting. In addition, pulmonary surfactant altered the antibiotic release: BDQ was not released, whereas LVX was (>80% in 24 h). The presence of mucus reduced the mobility of the liposomes. Globally, the antibiotic effect was preserved against *Mycobacterium abscessus* when the liposomes were deposited in the form of a dry-powder aerosol.

In 2022, Patil et al. studied the encapsulation of LZN in mannose-conjugated gelatin NPs of 200 nm–300 nm [142]. The drug entrapment was approximately 55%, and LZN was released over a period of 96 h in PBS, showing that the formulation was capable of sustained drug release. In vivo tests showed that mannose functionalization allowed to maintain drug concentrations above the MIC in the lungs of rats for a longer period of time compared to uncoated NPs and to free LZN. The analysis of the plasma content showed that the half-life of the drug was 19 times greater for encapsulated LZN compared to free LZN. After 28 days, no alterations were found in the biochemical and hematological parameters; liver, kidneys and hematological parameters suggested that the formulation was not toxic. These results support the idea of lowering the dose and frequency of administration of LZN with NP administration and, therefore, its associated toxicity.

**Figure 14:**
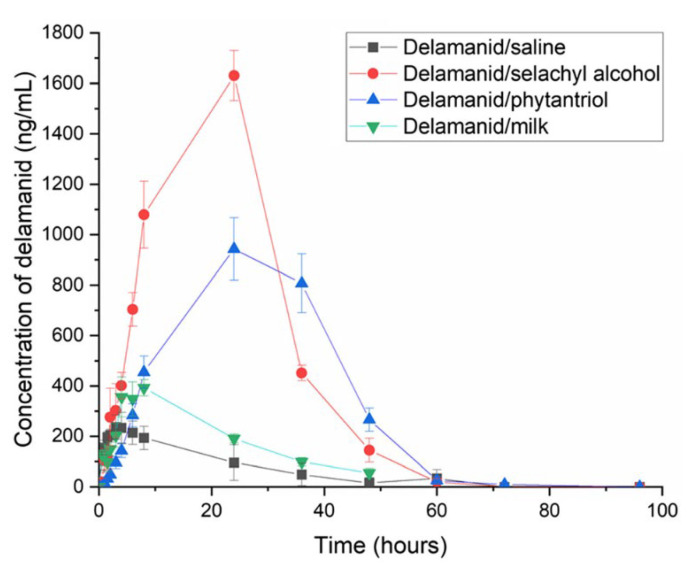
Concentrations of delamanid in plasma following oral administration to mice in aqueous saline suspension and through lipid-based formulations (phytantriol, selachyl alcohol and milk) over 96 h. *n* = 4. Adapted from reference [143], used under Creative Commons BY 4.0 license.

Since DLM and PMD are the most recently approved drugs, there is not much research on their encapsulation in nanocarriers yet. Here, we show two relevant examples. In 2021, Ramirez et al. loaded DLM onto nanostructures made of self-assembling lipids, selachyl alcohol and phytantriol [143]. These lipids formed liquid crystals endowed with high drug-loading capacities, and the ability to achieve controlled release (Figure 14). After oral administration in mice, the nanostructured lipid formulations extended the duration of absorption of DLM well beyond that from milk or suspension formulations.

In 2022, Ang et al. studied the encapsulation of PMD using mesoporous SiNPs [144]. The nanocarriers improved the solubility of the drug without compromising its effectiveness in killing *Mtb*. Additionally, a coating with amino groups improved the effectiveness after oral administration in mice.

**Table 2:** Overview of the publications dedicated to the encapsulation of second-line antitubercular drugs, ranked by active molecule and in chronological order. PdI: polydispersity index; drug encapsulation: quantity of encapsulated molecule compared to the quantity of molecule used during the preparation of nanovectors, expressed as a percentage; drug loading: mass of encapsulated molecule compared to the total mass of nanovectors, expressed as a percentage.

Physicochemical Properties	Biological Data	
Bedaquiline (BDQ)		
**Lipid NPs** [139]		
Preparation: ultrasonication Trimannose-conjugated NPs: Size: 83 nm to 86 nm PdI: <0.15 ζ potential: −10 mV or 28 mV Drug encapsulation: 93% Drug loading: 3%	In vitro	Drug release:BDQ-loaded NPs (28 mV): 75% in 14 h BDQ-loaded NPs (−10 mV): 95% in 14 h <10% of drug release after 7 days in PBS, RPMI and 7H9Effect against *Mtb*: Free BDQ, BDQ-loaded NPs (28 mV) and BDQ-loaded NPs (−10 mV): MIC of 0.03 µg/mL No toxicity towards THP-1 cells (below 1 mg/mL), HepG2 cells (below 1 mg/mL) and A549 cells (below 600 µg/mL)
	In vivo	Animal model: mouse Effect against *Mtb*: BDQ-loaded NPs: decrease in bacterial load after 13 days Strong accumulation in the lungs
**Chitosan NPs** [140]		
Preparation: nanoemulsion PEG-coated NPs: Size: 328 nm–456 nm PdI: 0.151–0.204 ζ potential: −9 mV Drug loading: 25% Non-coated NPs: Size: 328 nm–456 nm PdI: 0.151–0.204 ζ potential: 26 mV Drug encapsulation: 70% Drug loading: 28%	In vitro	Drug release: Coated NPs: >40% after 7 days in RPMI, <30% after 7 days in milli-Q water Non-coated NPs: 5% after 7 days in RPMI and in milli-Q water
	In vivo	-
**PLGA NPs** [145]		
Preparation: single emulsion Encapsulation of BDQ and Q203: Size: 480 nm PdI: 0.51 Drug encapsulation: BDQ: 55% Q203: 57% Combination: 41% for BDQ, 50% for Q203	In vitro	Drug release in simulated lung fluid: BDQ: 85% in 8 h Q203: 90% in 8 h Combination: 85% in 8 h for BDQ, 98% in 8 h for Q203 Abrupt drug release in 8 h, complete drug release in 24 h Effect against *Mtb*: BDQ: MIC50 of 120 nM Q203: MIC50 of 3 nM No toxicity towards A549 cells (below 500 µg/mL)
	In vivo	-
**Liposomes in lactose–leucine microcapsules** [141]		
Preparation: thin-film hydration and extrusion Size: 90 nm–100 nm PdI: <0.1 ζ potential: −14 mV Drug encapsulation: 98% Drug loading: 8%	In vitro	Drug release: No release in lung surfactant, <10% in milli-Q water
	In vivo	-
**Linezolid (LZN)**		
**Non-structured lipid carriers in mannitol–maltodextrin–leucine microparticles** [146]		
Preparation: hydration Size: 809 nm–820 nm PdI: 0.21–0.25 ζ potential: −58 mV–−37 mV Drug encapsulation: 96% Drug loading: 19% Microparticles size: 1.4 µm–2.5 μm	In vitro	Drug release (in PBS, for a pH of 7.4; in citrate buffer, for a pH of 4.5): LZN-loaded NPs: 32%–35% in 1 h, 85%–90% in 24 h No toxicity towards A549 cells
	In vivo	Animal model: mouse No toxicity 24 h after orotracheal administration compared to free LZN
**Gelatin NPs** [142]		
Preparation: desolvation Mannose-conjugated NPs: Size: 298 nm PdI: <0.148 ζ potential: 12 mV–27 mV Drug encapsulation: 51%–57%	In vitro	Drug release in PBS, for a pH of 7.4: LZN-loaded NPs: 95% in 96 h No toxicity towards J774 cells
	In vivo	Animal model: rat Bioavailability: Free LZN: detection in the plasma for up to 10 h–12 h LZN-loaded NPs: detection in the plasma for up to 3 days–5 days; 19-fold higher half-life compared to free LZN No toxicity after 28 days of repeated administrations
**PLGA NPs in microparticles** [147]		
Preparation: emulsion-solvent evaporation Size: 45 nm–178 nm Drug encapsulation: 57%–85% Microparticles size: 3.8 μm	In vitro	Drug release in simulated lung fluid: LZN-loaded NPs: 75%–90% in 120 h Effect against *Mtb*: Free LZN: MIC of 1 µg/mL LZN-loaded NPs: MIC of 0.6 µg/mL
	In vivo	-
**Chitosan NPs in microparticles** [148]		
Preparation: ionotropic gelation Size: 89 nm–223 nm Encapsulation efficiency: 37%–49% Microparticles size: 3.2 μm	In vitro	Drug release in simulated lung fluid: LZN-loaded NPs: 78%–90% in 24 h Effect against *Mtb*: Free LZN: MIC of 1 µg/mL LZN-loaded NPs: MIC of 0.8 µg/mL
	In vivo	-
**Ethionamide (ETH)**		
**Chitosan NPs** [149]		
Preparation: carrageenan-stabilized ionotropic gelation Size: 317 nm–324 nm PdI: 0.22–0.42 ζ potential: −13 mV–−24 mV	In vitro	Drug release: 0% of stabilizer: 95% in 24 h 42% of stabilizer: 95% in 24 h 59% of stabilizer: 80% in 24 h Effect against *Mtb*: Free ETH: MIC of 0.43 µg/mL ETH-loaded NPs: MIC of 0.61 µg/mL
	In vivo	-
**PLA NPs, PLGA NPs and CD-based NPs: ETH + BDM41906 (booster)** [127]		
PLA NPs: Preparation: nanoemulsion Size: 254 nm–277 nm PdI: <0.09 ζ potential: −5 mV Drug encapsulation: ETH: 76%–77% BDM41906: 46%–51% Drug loading: ETH: 36%–38% BDM41906: 23%–26% PLGA NPs: Preparation: nanoprecipitation Size: 170 nm Drug loading: <11% CD-based NPs: Preparation: nanoprecipitation Size: 10 nm Drug loading: ETH: 25 μg for 1 mg of NPs BDM41906: 25 µg for 1 mg of NPs	In vitro	Effect against *Mtb*: Free ETH and free BDM41906: IC50 of 0.11 µg/mL ETH- and BDM41906-loaded PLA NPs: IC50 of 0.06 µg/mL ETH- and BDM41906-loaded CD-based NPs: IC50 of 0.06 µg/mL
	In vivo	Animal model: mouse Effect against *Mtb*: ETH- and BDM41906-loaded CD-based NPs: 3-log reduction in CFUs in the lungs
**Codrug NPs: ETH + BDM43266 (booster)** [128]		
Preparation: nanoprecipitation of a codrug composed of ETH and of BDM43266 Size: 195 nm–208 nm Drug loading: 80%	In vitro	-
	In vivo	Animal model: mouse Effect against *Mtb*: ETH- and BDM43266-loaded NPs: 6-fold reduction in CFUs in the lungs

### 4.8. Host-Directed Therapy Using Nanoparticles

As previously stated, most human hosts are able to contain TB infection and avoid its progression towards active TB through the expression of a balanced, homeostatic immune response. Pro-inflammatory mechanisms that aim to kill, slow the progression and sequester *Mtb* are key to a successful host response [150]. However, if the pro-inflammatory response is excessive or inappropriate, tissue damage or granuloma enlargement might occur. The host also expresses a series of anti-inflammatory mediators which may be either beneficial or detrimental, depending on the timing of their secretion.

The aim of host-directed therapy is to increase the success of TB treatment by providing immunomodulation to the host response to infection. This is achieved by interfering with the host’s mechanisms required by *Mtb* for its persistence and replication [151]. Without interacting with *Mtb* itself, host-directed therapy aims to enhance the host defense mechanisms against *Mtb* by targeting the pathways perturbed by this pathogen. Autophagy is an important cellular process leading to the destruction of invading pathogens such as *Mtb* [152]. NPs were used for inducing autophagy activation to destroy intracellular *Mtb*. Several strategies were proposed, based on repurposing already approved drugs, vitamins and cytokines, alone or in conjunction with existing TB drugs [153]. Host-directed therapy allowed increasing the production of ROS (reactive oxygen species), the synthesis of antimicrobial peptides, the autophagy in infected cells and reducing inflammation and tissue damage during the active stage of the disease.

As the host-directed therapy acts on the host immune response rather than on *Mtb* directly, resistance issues are not a concern, which is a major advantage. In addition, NPs can be used to stimulate macrophages into a bactericidal state to eliminate intracellular *Mtb*. For instance, the polysaccharide glucan stimulates macrophages to produce pro-inflammatory signals (ROS and reactive nitrogen species), including IL-12 and TNFα, which are known to be crucial in the control of *Mtb* [154,155]. More recently, it was shown that curdlan-loaded PLGA NPs with sizes between 330 nm and 453 nm significantly upregulated the pro-inflammatory cytokine TNF-α [156]. As a consequence, the NPs reduced the intracellular *Mtb* burden over 72 h in infected RAW264.7 macrophages.

In a recent study, PLGA NPs were loaded with all-*trans*-retinoic acid and tested with a commercially available nebulizer generating droplets of NP suspension with a mass median aerodynamic diameter of approximately 2 µm–4 μm [157]. These NPs, suitable for nebulization, were able to reduce *Mtb* growth in THP-1-derived macrophages. In a breathing simulation experiment, 65% of the dose of drug-loaded NPs was inhaled.

An NP formulation encapsulating curcumin, a compound with anti-IL-10 activity, was tested in a murine TB model [158]. IL-10 is a key anti-inflammatory cytokine which suppresses T-cell function, blunts inflammatory responses, and promotes TB progression. The NP formulation showed modest activity as a monotherapy, but more potent activity in combination with INH.

Host defense peptides represent an alternative to classical therapeutics with lesser susceptibility of resistance. Synthetic magainin-I analog peptide (MIAP) was encapsulated to increase its stability and administered to the lungs under the form of a micron-sized dry powder [159]. These MIAP-loaded nano-assemblies demonstrated dose- and time-dependent antibacterial activity against virulent *Mtb* for at least 96 h, with up to a 3-log CFUs reduction in viable bacteria as compared to the untreated group. The host defense mechanism was improved by averting the bacteria-induced inhibition of phagosomal-lysosome fusion and apoptosis.

Glucan NPs loaded with antitubercular drugs displayed a strong innate immune response in *Mtb*-infected macrophages, including the production of ROS and reactive nitrogen species, autophagy and apoptosis [160]. The formulation not only activated *Mtb*-infected and immune-suppressed macrophages for host-directed therapy, but also increased the effectiveness of the loaded drug (rifabutin) by 2.5-fold. 

PLGA NPs loaded with N-acetyl-L-cysteine and delivered to the lungs acted as host-directed therapies, with better antibacterial activity than the free drug against *Mtb* [161]. Advantageously, N-acetyl-L-cysteine has antioxidant, mucolytic, anti-inflammatory and antimycobacterial effects by enhancing interleukins and INF-γ production [162].

### 4.9. Combined Therapies to Treat Tuberculosis

**Figure 15:**
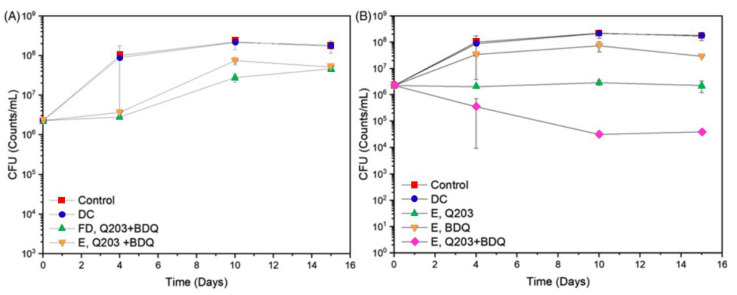
Effect of bedaquiline- (BDQ) and Q203-loaded polymeric nanoparticles (NPs) on bacillus Calmette–Guérin. The colony forming unit (CFU) count with respect to the incubation time is shown. (**A**) Comparison when drugs are free (FD) or encapsulated (E). (**B**) Comparison when drugs are encapsulated (E) separately or together. DC: drug carrier (empty NPs). Adapted from reference [145], used under Creative Commons BY 4.0 license.

The use of magnetic NPs is one of the new strategies for the treatment of TB. In 2019, Poh et al. investigated the co-encapsulation of BDQ and Q203 along with supermagnetic iron oxide NPs into 500 nm PLGA NPs [145]. Q203 is a promising first-class candidate against *Mtb* which is currently under phase II clinical trials [163]. Since Q203 does not have cross-resistance with BDQ and both drugs target the same cell structure, their combined synergistic effects are a promising alternative for the MDR-TB treatment. The incorporated iron oxide NPs enabled one to direct the PLGA NPs to a specific site through the use of magnets. In vitro studies showed that encapsulated drugs were as effective as free drugs against BCG. Interestingly, BDQ and Q203 separately failed to lower the amount of CFUs in in the lungs; however, when both drugs were co-encapsulated, a 2-fold decrease in CFUs was observed (Figure 15).

Besides previously cited magnetic guidance, sonodynamic antibacterial chemotherapy was proposed as a methodology to eliminate bacteria and to treat MDR-TB [164]. LVX-loaded PLGA-PEG NPs were conjugated with a BM2 aptamer to target BCG in an *Mtb*-infected rat model, in the presence of ultrasound stimulations. The NPs were specifically recognized by BCG in vitro and accumulated in the lesion tissue. Moreover, the loaded drugs with ultrasound-responsive properties were effectively released. In a nutshell, the functionalized PLGA NPs exhibited a significant sonodynamic efficiency and produced high ROS amounts, resulting in an efficient bacterial elimination in vitro. In vivo, the NPs showed excellent ultrasound therapeutic effects in a BCG-infected rat model. The PLGA NPs containing LVX could effectively transfer the therapeutic drugs into cells and improved the bactericidal effect under ultrasound.

Similarly, LVX-loaded PLGA NPs were prepared by a double emulsification method, and their antibacterial activity against *Mycobacterium smegmatis* residing inside macrophages was tested in conjunction with low-frequency and low-intensity ultrasound [165]. The ultrasound irradiation at 42 kHz with an intensity of 0.13 W/cm^2^ for 10 min significantly promoted the phagocytosis of the NPs by the macrophages. In addition, further ultrasound combined with the LVX-loaded NPs promoted the production of ROS in macrophages, increasing their apoptosis rate. Ultrasound combined with the LVX-loaded NPs exhibited a 10-fold higher antibacterial activity against *Mycobacterium smegmatis* residing inside macrophages compared to the free drug.

Recently, MOFs based on zirconium ions and porphyrin ligands were loaded with a synthetic oligodeoxyribonucleotide sequence able to induce a Th1-type immune response by stimulating Toll-like receptors in mammalian immune cells, in an attempt to eradicate *Mtb* [166]. In addition, porphyrin ligands are photosensitizers allowing the NPs to play a role in photodynamic therapy while delivering their drug cargo. Phosphatidylserine is a compound abundant in the outer membrane of apoptotic cells. It plays a major role in the recognition and phagocytosis of apoptotic cells by macrophages. Therefore, to target the sanctuary of *Mtb*, the NPs were functionalized with phosphatidylserine. The resulting bio-inspired targeted DDS had a bactericidal activity in vitro and the ability to stimulate the immune system in vivo.

### 4.10. Summary of the Output of Nanoparticles to Treat Tuberculosis

**Figure 16:**
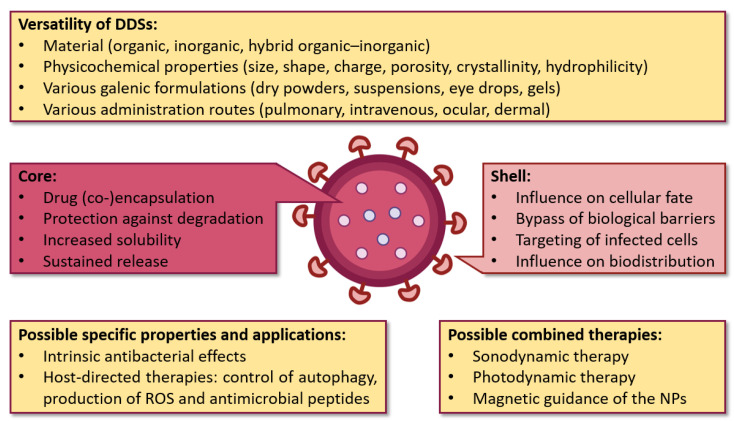
Schematic summary of the advantages and properties of nanosized drug delivery systems (DDSs) for the treatment of tuberculosis. Drug-loaded nanoparticles (NPs) can be administered by various routes, and their surface can be engineered with functional coatings. They can also be endowed with intrinsic antibacterial effects. Possible applications are in the field of host-directed or combined therapies. ROS: reactive oxygen species. Created with BioRender.com.

In conclusion, it appears that the different properties of NPs constitute a virtuous circle (Figure 16). Whatever the evolution of the physiopathology of *Mtb,* the pathogen can be tracked using engineered NPs: in the lungs (in the case of pulmonary TB), in the bones and in other organs (in the case of extra-pulmonary TB), and finally, in the whole organism (in the case of miliary TB). Thus, the vectorization of active molecules opens the way for personalized treatments, tailor-made for each patient, based on various and adapted galenic formulations (such as powders for inhalation, suspensions for sprays or for oral administration, injections and eye drops).

Furthermore, targeting a preferential location in the organism (sometimes, even down to the cellular level) enables one to increase the bioavailability of the active molecule while reducing the administered doses. Thus, the benefit is two-fold: firstly, concentrating the antibacterial effect in a site of interest enables one to optimize the efficacy of the treatment, and secondly, reducing the doses results in the diminution of the side effects. NPs also enable controlled and prolonged drug release, which allows one to space out the administered doses, for a therapy which is just as effective as the standard treatment. Other advantages of NPs are a dramatically increased transit through the mucus barrier, low adhesion to lung mucus, disrupting the bacterial biofilm and providing uniform drug delivery to the lungs after pulmonary delivery [167].

In summary, a therapy based on NPs would reduce the treatment toxicity and complexity, which would ensure considerable progress in terms of patient compliance. Since the main source of antibiotic resistance are relapses caused by incomplete therapy, NPs could ultimately enable the successful treatment of most patients, and therefore, reach the next stage in the fight against *Mtb*.

## 5. Conclusions and Perspectives

The objective here was to give an overview of how nanotechnologies could improve the treatment of TB. Clearly, this topic attracts growing interest, as evidenced by the hundreds of publications dedicated to the subject. In contrast, the transition of the NPs to the clinical stage is not achieved yet. In the introduction, a relatively modest amount (about fifty) of nanoformulations currently involved in clinical trials was mentioned, which are for all diseases, but there were no candidates yet for TB [168]. Of note, several nanoformulations are currently going through clinical trials for the treatment of COVID-19 (clinicaltrials.gov, accessed on 11 January 2023). Current research for the treatment of TB seems to be more focused on the discovery of new active molecules [169] and new vaccines (some of which, nevertheless, contain NPs) [170,171]. However, in the last twenty years, only four antitubercular drugs with novel mechanisms of action have been approved to treat MDR/XDR-TB: LZN, BDQ, DLM and PMD.

Which are the reasons for such a contrast between the profusion of publications dedicated to NPs for the treatment of TB, and their still tenuous impact regarding clinical trials? This review yet demonstrates that the potential of NPs is promising and could truly transform the antitubercular therapy. However, several factors hamper the transition from the research laboratory to the patient. Firstly, the largest number of publications are only dedicated to the development of new formulations, without the in vivo proof of concept, a step that is nevertheless necessary to assess the therapeutic potential of the studied formulations. In vivo studies were mostly carried out in models of infected mice and rats, rarely rabbits [172]. However, in vivo testing using animal TB models is time-consuming, costly, and represents a major bottleneck in drug nanocarrier discovery and development. To avoid using mammals, screening studies on zebrafish embryo were also proposed [173]. The zebrafish TB model emerged as a quick and sensitive tool for evaluating the in vivo toxicity and efficacy of drug formulations in their early stages of development.

Preclinical studies require an interdisciplinary approach and expertise in many fields, including chemistry, nanotechnology and biology. At the chemical level, the complexity of certain NP formulations is such that several years are sometimes necessary to engineer an optimized drug nanocarrier. At the biological level, research on infected animal models is financially very demanding and requires costly and complex infrastructures (high-security laboratories). Thus, the in vivo proof of concept cannot be carried out without significant financial means and collaborations between laboratories. In addition to safety and toxicity assessments, as well as regulatory aspects, it will take years before a new formulation is approved.

The transition from experimental research to clinical trials requires an extensive understanding of the studied NPs’ fate, both in vitro and in vivo. However, TB is a complex pathology which is not easy to model in vitro. For instance, recreating the structure of a granuloma is a complex topic [174]. Different models were proposed, some enabling one to induce latency and reactivate *Mtb*. Organs-on-chips simulate the alveolar-capillary interface using cell cocultures, and can even mimic the phenomenon of respiration through a system which stretches a membrane at regular intervals. However, these models have limitations, including the difficulty to mimic in a laboratory the complexity of the lung fluid and microenvironment.

Furthermore, ensuring the colocalization between the active molecule and the pathogen at the intracellular level is one of the biggest challenges. Indeed, depending on their size, shape and surface properties, NPs localize in different cell compartments, and the released drug may have to bypass intracellular membranes to reach the pathogen (for example, to cross the lysosomal membrane to attain *Mtb* in the cytosol) [175]. Labeling NPs to study their internalization within macrophages can be successfully achieved [176]. Nevertheless, labeling the active molecule itself to study its release from NPs presents the risk of modifying its physicochemical properties and activity, and thus its intracellular fate in an unknown manner.

Studies in the literature with antitubercular drug nanocarriers mostly focus on SLNPs as well as polymeric NPs (PLGA, chitosan, alginate), allowing the co-encapsulation of several first-line antibiotics, and the considerable reduction in the treatment posology [177,178]. To date, only scarce studies have dealt with the encapsulation of second-line drugs, and especially newly discovered active molecules such as LZN, BDQ, DLM and PMD. With the increase in MDR/RR-TB cases, it is expected that more investigations will be dedicated to the encapsulation of these promising drugs.

Metal- and CD-based NPs stand out for an optimized treatment of TB, as they possess intrinsic antibacterial properties allowing a synergistic action between the active molecules and their nanocarrier. In addition, host-directed therapy using NPs is a promising new approach [153]. For instance, novel innovations in antitubercular therapeutics have been envisaged based on manipulating autophagy activation, such as NPs encapsulating conventional antitubercular drugs and autophagy-inducing compounds capable of host-directed therapies. Autophagy is a cellular process responsible for the lysosomal degradation of intracellular components, including invading *Mtb*. Thus, the manipulation of autophagy activation in the infected host cell using autophagy-inducing compounds has attracted significant interest as an alternative new approach for treating TB [179]. Another challenge of host-directed TB therapy is to prevent the irreversible lung damage caused by an ineffective inflammatory response. Delivering, by means of NPs, compounds that have the potential to minimize non-productive inflammation and potentially severe tissue damage, is appealing.

Perhaps, in view of the recent discoveries concerning the SARS-CoV-2 pandemic, the next innovation for the treatment of *Mtb* will consist of an mRNA vaccine comprising NPs. More than one hundred years ago, Paul Ehrlich dreamed about a “magic bullet” able to specifically reach a diseased site in the organism, minimizing the toxicity in healthy tissues. Possibly, engineered NPs will be able to answer the problematics linked to TB treatment by delivering a synergistic drug cocktail directly to the infected lungs and to other diseased organs. Maybe new powerful active molecules will be integrated into the therapeutic arsenal. Scientists’ imagination is borderless, and the possibilities are numerous; nanomedicine may be on the verge of stunning new discoveries.

## Data Availability

Publicly available datasets were analyzed in this review for Figure 7. This data can be found here: www.who.int/tb/data.

## Abbreviations

AuNP	gold nanoparticle
BCG	bacillus Calmette–Guérin
BDM	family of molecules boosting the activity of ethionamide
BDQ	bedaquiline
CD	cyclodextrin
CFU	colony forming unit
CFZ	clofazimine
DDS	drug delivery system
DLM	delamanid
EMB	ethambutol
ETH	ethionamide
INH	isoniazid
INP	inorganic nanoparticle
LVX	levofloxacin
LZN	linezolid
MDR-TB	multidrug-resistant tuberculosis
MOF	metal–organic framework
MOX	moxifloxacin
*Mtb*	*Mycobacterium tuberculosis*
NP	nanoparticle
PCL	poly(ε-caprolactone)
PEG	polyethylene glycol
PLA	poly(lactic acid)
PLGA	poly(lactic-*co*-glycolic acid)
PMD	pretomanid
PNP	polymeric nanoparticle
pre-XDR-TB	pre-extensively drug-resistant tuberculosis
PZA	pyrazinamide
RFP	rifampicin
RR-TB	rifampicin-resistant tuberculosis
SiNP	silica nanoparticle
SLNP	solid lipid nanoparticle
TB	tuberculosis
XDR-TB	extensively drug-resistant tuberculosis
